# Preconception health and care policies, strategies and guidelines in the UK and Ireland: a scoping review

**DOI:** 10.1186/s12889-024-19188-0

**Published:** 2024-06-22

**Authors:** Emma H. Cassinelli, Michelle C. McKinley, Lisa Kent, Kelly-Ann Eastwood, Danielle A. J. M. Schoenaker, David Trew, Theano Stoikidou, Laura McGowan

**Affiliations:** 1 Centre for Public Health (Institute for Global Food Security), School of Medicine, Dentistry and Biomedical Sciences, Queen’s University Belfast, Belfast, UK; 2 University Hospitals Bristol NHS Foundation Trust, Bristol, UK; 3 School of Human Development and Health, Faculty of Medicine, University of Southampton, Southampton, UK; 4 MRC Lifecourse Epidemiology Centre, University of Southampton, Southampton, UK; 5 NIHR Southampton Biomedical Research Centre, University of Southampton and University Hospital Southampton NHS Foundation Trust, Southampton, UK; 6 Patient and Public Involvement and Engagement “Healthy Reproductive Years” Panel, Belfast, UK

**Keywords:** Preconception health, Preconception care, Pre-pregnancy, Healthcare, Scoping review, Grey literature, Content analysis, Audit

## Abstract

**Background:**

Preconception health has the potential to improve parental, pregnancy and infant outcomes. This scoping review aims to (1) provide an overview of the strategies, policies, guidelines, frameworks, and recommendations available in the UK and Ireland that address preconception health and care, identifying common approaches and health-influencing factors that are targeted; and (2) conduct an audit to explore the awareness and use of resources found in the scoping review amongst healthcare professionals, to validate and contextualise findings relevant to Northern Ireland.

**Methods:**

Grey literature resources were identified through Google Advanced Search, NICE, OpenAire, ProQuest and relevant public health and government websites. Resources were included if published, reviewed, or updated between January 2011 and May 2022. Data were extracted into Excel and coded using NVivo. The review design included the involvement of the “Healthy Reproductive Years” Patient and Public Involvement and Engagement advisory panel.

**Results:**

The searches identified 273 resources, and a subsequent audit with healthcare professionals in Northern Ireland revealed five additional preconception health-related resources. A wide range of resource types were identified, and preconception health was often not the only focus of the resources reviewed. Resources proposed approaches to improve preconception health and care, such as the need for improved awareness and access to care, preconceptual counselling, multidisciplinary collaborations, and the adoption of a life-course approach. Many behavioural (e.g., folic acid intake, smoking), biomedical (e.g., mental and physical health conditions), and environmental and social (e.g., deprivation) factors were identified and addressed in the resources reviewed. In particular, pre-existing physical health conditions were frequently mentioned, with fewer resources addressing psychological factors and mental health. Overall, there was a greater focus on women’s, rather than men’s, behaviours.

**Conclusions:**

This scoping review synthesised existing resources available in the UK and Ireland to identify a wide range of common approaches and factors that influence preconception health and care. Efforts are needed to implement the identified resources (e.g., strategies, guidelines) to support people of childbearing age to access preconception care and optimise their preconception health.

**Supplementary Information:**

The online version contains supplementary material available at 10.1186/s12889-024-19188-0.

## Background

Preconception health describes the overall health of non-pregnant individuals of childbearing age (15–49 years) [1], and preconception care is defined as “the provision of biomedical, behavioural and social health interventions to women and couples before conception occurs” [2]. Preconception care represents an excellent opportunity for the identification, screening, management and prevention of preconception health risk factors including, for example, parental obesity, long-term physical and mental health conditions, alcohol consumption, smoking, physical inactivity, inadequate dietary habits, poor social support and low immunisation levels [3–6]. These preconception risk factors are widespread across the population [7], and individuals often live with more than one risk factor concurrently [8]. The optimisation of preconception health provides an opportunity to improve individuals’ wellbeing and promote positive intergenerational health, given the well-recognised association between parental and child health [9, 10].

There has been an increased global recognition of the importance of preconception health and care in recent years [6], and reviews of preconception-focused guidelines and policies have been previously published. For example, an investigation of preconception policies, guidelines, recommendations and services was conducted across six countries, including the UK, and found heterogeneity in the advice provided [3]. Given that these findings are related to searches conducted in 2013, a renewed investigation spanning the past decade was warranted and justified. Since then, a further review on preconception guidelines, recommendations and policy reports has been carried out [6]. This review aimed to inform the reporting of population-level preconception health in England and led to the development of a comprehensive list of preconception indicators [6]. A recent systematic review explored international clinical guidelines on preconception care, however it did not include guidelines specific to the UK or Ireland [11].

These previous reviews have played a critical role in advancing our understanding of preconception health and care. However, as the field of preconception care continues to grow and progress, it is important to collect, summarise and evaluate available contemporary resources. To the best of our knowledge, no previous scoping review has identified and comprehensively described the content of preconception strategies, policies, guidelines, frameworks and recommendations in the UK and Ireland. There is also limited knowledge on healthcare professionals’ (HCPs) awareness and use of preconception health and care resources.

### Research aims and objectives

This scoping review aims to provide insights for HCPs and policymakers, with the aim of improving preconception care delivery. Two primary objectives were established: (1) to conduct a scoping review to offer an overview of preconception health and care strategies, policies, guidelines, frameworks and recommendations from the UK and Ireland, and identify the common approaches and health-influencing factors addressed; and (2) to conduct an audit to validate available resources in Northern Ireland. The specific research questions this piece of work aims to answer can be found in Table 1.

**Table 1:** Research questions

1. What strategies, policies, guidelines, frameworks and recommendations have been developed that address preconception health and care for adults in the UK and Ireland between January 2011 and May 2022? 2. How does the evidence from strategies, policies, guidelines, frameworks and recommendations that address preconception care for adults differ across the UK and Ireland? 3. What are the main concepts and themes underpinning strategies, policies, guidelines, frameworks and recommendations that address preconception health and care for adults in the UK and Ireland? 4. What are, if any, the gaps in the knowledge provided in strategies, policies, services, guidelines, frameworks and recommendations that address preconception health and care for adults in the UK and Ireland, and what areas require further coverage and inquiry? 5. What are, if any, the services and interventions provided in Northern Ireland focused on improving preconception health and care in adults?	

## Methods

A detailed research protocol has been previously published [12], with the main methodology summarised below.

This scoping review was conducted in accordance with the Joanna Briggs Institute’s updated methodological guidance for scoping reviews [13] and Arksey and O'Malley’s framework for conducting scoping studies [14]. Reporting was informed by the Preferred Reporting Items for Systematic Reviews and Meta-analyses extension for scoping reviews (PRISMA-ScR; see Additional file 1) [15].

Ethical approval was not required as the review analysed content from publicly accessible resources and the case study in Northern Ireland was conducted for audit purposes.

### Search strategy

Searches were carried out on Google Advanced Search, National Institute for Health & Clinical Excellence (NICE), OpenAire, ProQuest and relevant public health and government websites using words and phrases such as “preconception health”, “preconception care”, “pre-pregnancy” and “preparation for pregnancy” (see Additional files 2 and 3 for full details).

### Inclusion and exclusion criteria

The inclusion and exclusion criteria adopted are shown in Tables 2 and 3.

**Table 2:** Inclusion criteria for resource selection

• Grey literature resources including strategies, policies, guidelines, frameworks and recommendations such as leaflets, booklets, webpages, and e-learning courses, discussing or addressing preconception health and care for individuals of childbearing age • Resources from the UK and Ireland • Resources written in the English language • Resources published, reviewed or updated between January 2011 and May 2022, thereby building upon the timeframe of a previous review [3] and allowing for more than a decade of content to be assessed • Resources providing specific advice, offering recommendations, or outlining policy actions or strategic plans to improve preconception health and care for individuals of childbearing age

**Table 3:** Exclusion criteria for resource selection

• Resources not identified as grey literature, including journal articles, preprints, working papers from research groups, visual or audio content, academic letters or commentaries, calls for participants, presentations and doctoral dissertations • Resources not addressing preconception health and care for individuals of childbearing age, including resources explicitly addressing only the interconception period • Resources from countries other than the UK and Ireland • Resources not written in the English language • Resources published, reviewed or last updated before January 2011 • Resources not providing specific advice, recommendations, policy actions or strategic plans to improve preconception health and care for individuals of childbearing age, thereby lacking sufficient detail and depth of advice (e.g., resources solely mentioning preconception health or signposting other material)	

### Data extraction and analysis

Identified resources were collated and uploaded into Microsoft Excel, duplicates were removed, and titles and summaries were screened. Following the methodology used by Godin and colleagues [16], the first 100 results on Google Advanced Search were screened for potentially relevant titles. The full texts of potentially relevant citations were assessed in detail, with ≥ 10% of resources being double-coded for inclusion in the review based on inclusion and exclusion criteria (EHC, LM). Any disagreements between the reviewers during the selection or coding process were resolved through discussion with the wider research team until consensus was reached, achieving multidisciplinary triangulation (disciplines included public health, psychology, and nutrition). NVivo 20 was used to manage the coded data and perform the content analysis (e.g., the identification of themes).

The term “resource” will be used throughout to indicate any retrieved record, including policies, strategies, guidelines, frameworks and recommendations.

### Audit – Northern Ireland

An audit was undertaken in Northern Ireland to validate and contextualise findings from the scoping review, confirm the breadth of coverage and identify other potentially eligible resources. The audit was carried out through the dissemination of a brief checklist, exploring stakeholders’ awareness and use of identified resources (see Additional file 4). It aimed to involve stakeholders working in the healthcare system in Northern Ireland, relevant services and organisations (e.g., maternity service providers, midwives, gynaecologists, obstetricians, general practitioners, nurses, pharmacists). Contacts of the research team, word of mouth and findings from Google Advanced Search were used to identify and invite stakeholders to participate. Stakeholders received the checklist via email, together with background information on the review. A smaller subset of stakeholders was asked to pilot the checklist and provide feedback before it was refined and distributed more widely.

### Patient and Public Involvement and Engagement

Active involvement of the Patient and Public Involvement and Engagement (PPIE) panel “Healthy Reproductive Years” was achieved throughout the study. This advisory panel includes *n* = 23 adults aged 18–45 years old and living in Northern Ireland at the time of recruitment. Most of the panel members are female (*n* = 21) and did not have children at the time of recruitment (*n* = 11). Other demographic factors, such as ethnicity, were not collected. The panel members were recruited via numerous avenues including social media (e.g., Facebook) and relevant organisations (e.g., Sure Start). The PPIE strategies aimed to engage the public as partners [17], stimulate general discussions on preconception health and care to support priority setting, and advise on the research design and scoping review protocol [12], interpretation of results (including the terminology used and summary infographic), and dissemination plans. Engagement was carried out online (i.e., three workshops, exchange of emails), and representatives were financially remunerated in line with guidance from the National Institute for Health and Care Research [18]. They were sent information about relevant PPIE training to further develop their skills and understanding of involvement in research, and a regular newsletter shared updates about the study’s progression.

To report PPIE strategies, the Guidance for Reporting Involvement of Patients and the Public (GRIPP) 2 short form checklist was used [19] (see Additional file 5).

### Terminology

The present review used the term “woman” throughout. This should be taken to include people who do not identify as women [20] but may become pregnant. The term was chosen to mirror the language used in a previous review, on which the present work builds on [3], and in the majority of the reviewed content, including resources from NICE (e.g., [20, 21]). We acknowledge that others may prefer to use different terminology and that the preferred words may undergo further modifications in the coming years in this evolving field of study.

## Results

**Fig. 1:**
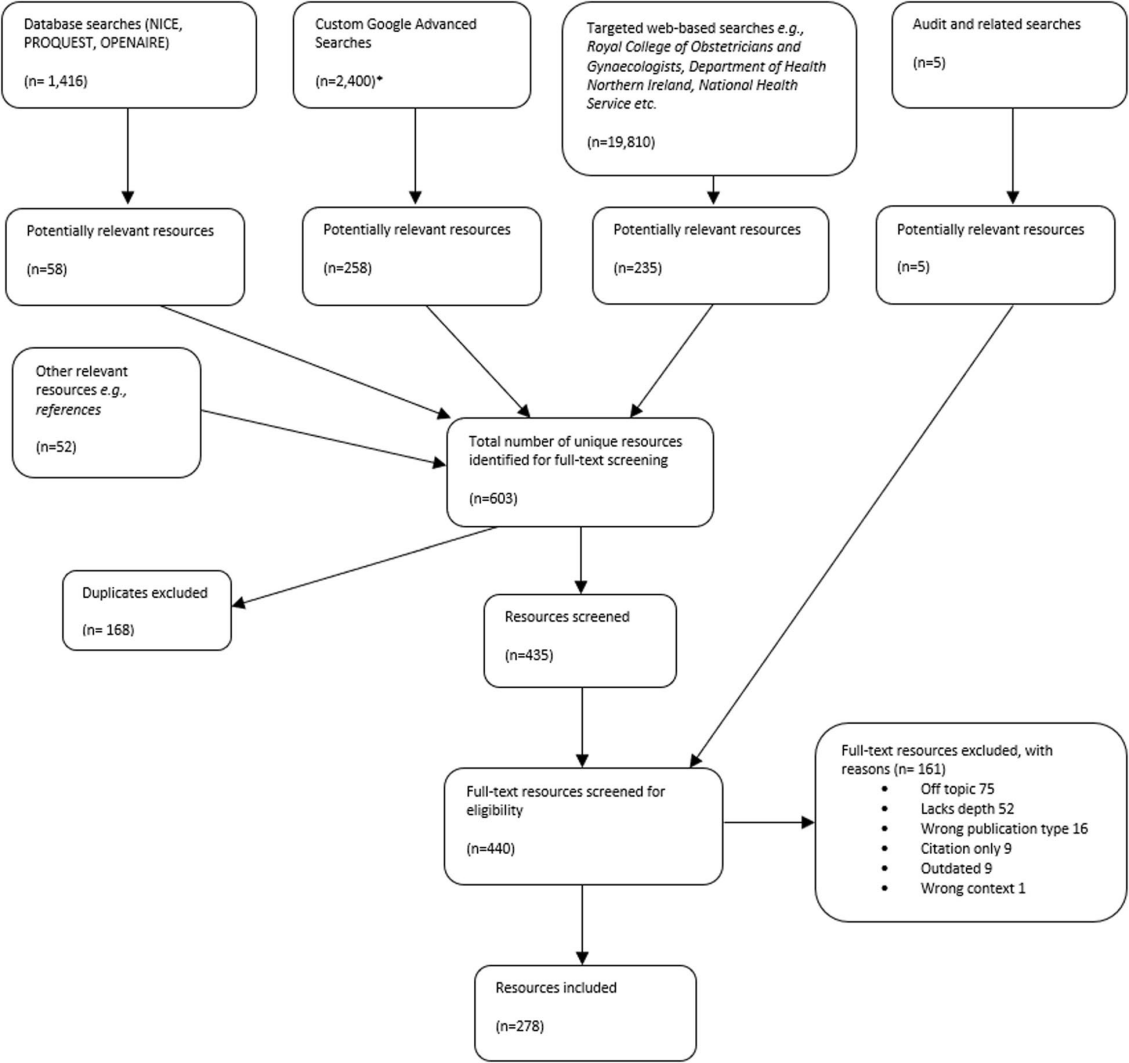
PRISMA flowchart. *Only the first 100 results for each Google Advanced Search searches were screened, following the methodology presented by Godin et al. [16]

This scoping review aimed to provide an overview of strategies, policies, guidelines, frameworks and recommendations addressing preconception health and care in the UK and Ireland. The searches were carried out in May 2022. After screening titles and summaries, 435 unique and potentially relevant resources were identified. Through audits with HCPs in Northern Ireland, five additional resources were identified, resulting in a total of 440 full-text resources for screening. Guided by the inclusion criteria (Table 2), 278 resources were included (Fig. 1; Additional file 6).

### Overview of resources identified (Research question 1, Research question 2)

A wide range of resources addressing preconception health and care were identified and included. Specifically, the review included *n* = 25 policies, strategies, action plans, manifestos, interventions and frameworks, *n* = 6 e-learning resources, and *n* = 118 reports, guidelines, statements and toolkits, mainly targeting a professional audience. Additionally, there were *n* = 84 websites and *n* = 45 recommendations, which comprised leaflets and booklets designed for public use. There was overlap between the categories, meaning that, for example, a single resource could have been categorised as both a report and a strategy. Approximately half of the resources reviewed (*n* = 128, 46%) were designed for public usage (e.g., leaflets, websites), primarily targeting women. The remaining resources (*n* = 150, 54%) were directed at specific professional audiences (e.g., HCPs, service commissioners, policymakers, governmental departments), occasionally also targeting the public. When stakeholder engagement was mentioned in resources, this included audiences such as service users and the public. Men and partners were directly mentioned in 37.1% (*n* = 103) of resources.

Results showed that prior to 2015 only a limited number of resources addressing preconception health or care were published (< 10 per year). Since then, the availability of resources has increased. In 2021, the highest number of resources was made available (*n* = 52).

When assessing the input from each individual country, Wales was the country with the lowest number of included resources specific to the country (*n* = 11), while Ireland had the highest (*n* = 50). Most resources (*n* = 119) were relevant to all countries in the UK (i.e., England, Scotland, Wales and Northern Ireland).

In terms of the evidence sources referenced in the resources, frequent citations included NICE, Royal colleges such as the Royal College of Obstetricians and Gynaecologists and the Royal College of Psychiatrists, peer-reviewed journal articles and the National Health Service (NHS). Other references included Public Health England (now replaced by the UK Health Security Agency and Office for Health Improvement and Disparities) and charities such as Tommy’s. There was cross-referencing between countries in the UK and Ireland, and certain resources also referenced international resources (e.g., from the United States). Many of the resources directed at the public, such as leaflets and websites, did not include reference lists or clear information on the sources of the evidence presented in the sections focusing on preconception health and care (*n* = 111, 39.2%).

### What were the main themes identified? (Research question 3)

**Fig. 2:**
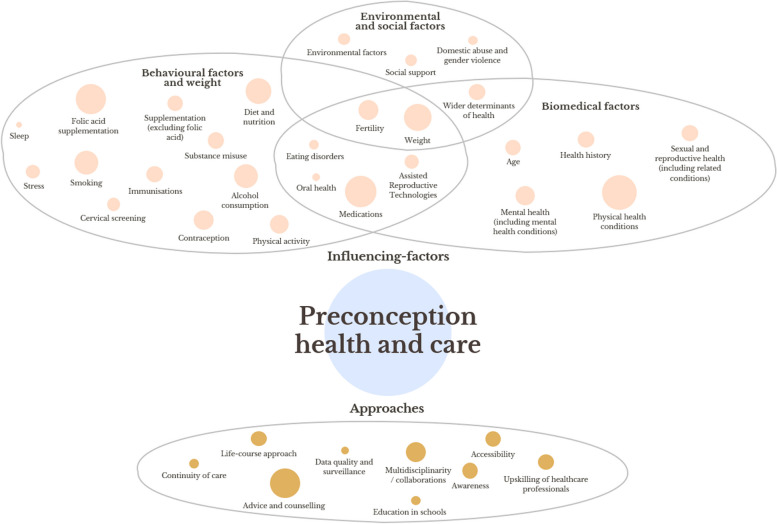
Commonly-proposed approaches to improve preconception health and care and preconception health-influencing factors

The content analysis of the included resources led to the identification of *n* = 36 themes, covering both commonly-proposed approaches to improve preconception health and care delivery (*n* = 9), and protective and risk factors that can influence preconception health (*n* = 27) (Fig. 2, where the size of nodes reflects the frequency of mentions). The results pertaining to the proposed approaches, which ultimately refer to actions that can help shape relevant services in the future, were presented mostly in resources for a professional audience, whereas health influencing factors were discussed in resources for both professionals and the public.

#### Approaches to improve preconception health and care

The importance of providing preconception advice and counselling was emphasised in 45% of the available resources (*n* = 125). Recommendations to improve the delivery of advice included a) avoiding overwhelming people by providing excessive information in a single instance; b) avoiding apportioning blame or guilt; c) including men, partners, family members or peers when accepted and appropriate; d) acknowledging societal and cultural factors, unconscious bias, and any barrier that may prevent the adoption of preconception health-promoting behaviours (e.g., financial circumstances, personal experiences, lack of support, and physical, sensory, cognitive or learning disabilities); and e) adopting a blended format (e.g., a single leaflet could combine messages on both folic acid supplementation and pre-pregnancy weight) [22–28]. Appropriate settings for the delivery of preconception care mentioned included both medical (e.g., pharmacies, doctor surgeries, family planning clinics, abortion and fertility clinics) and non-medical settings (e.g., community and youth centres, faith groups, hostels) [4, 25].

**Fig. 3:**
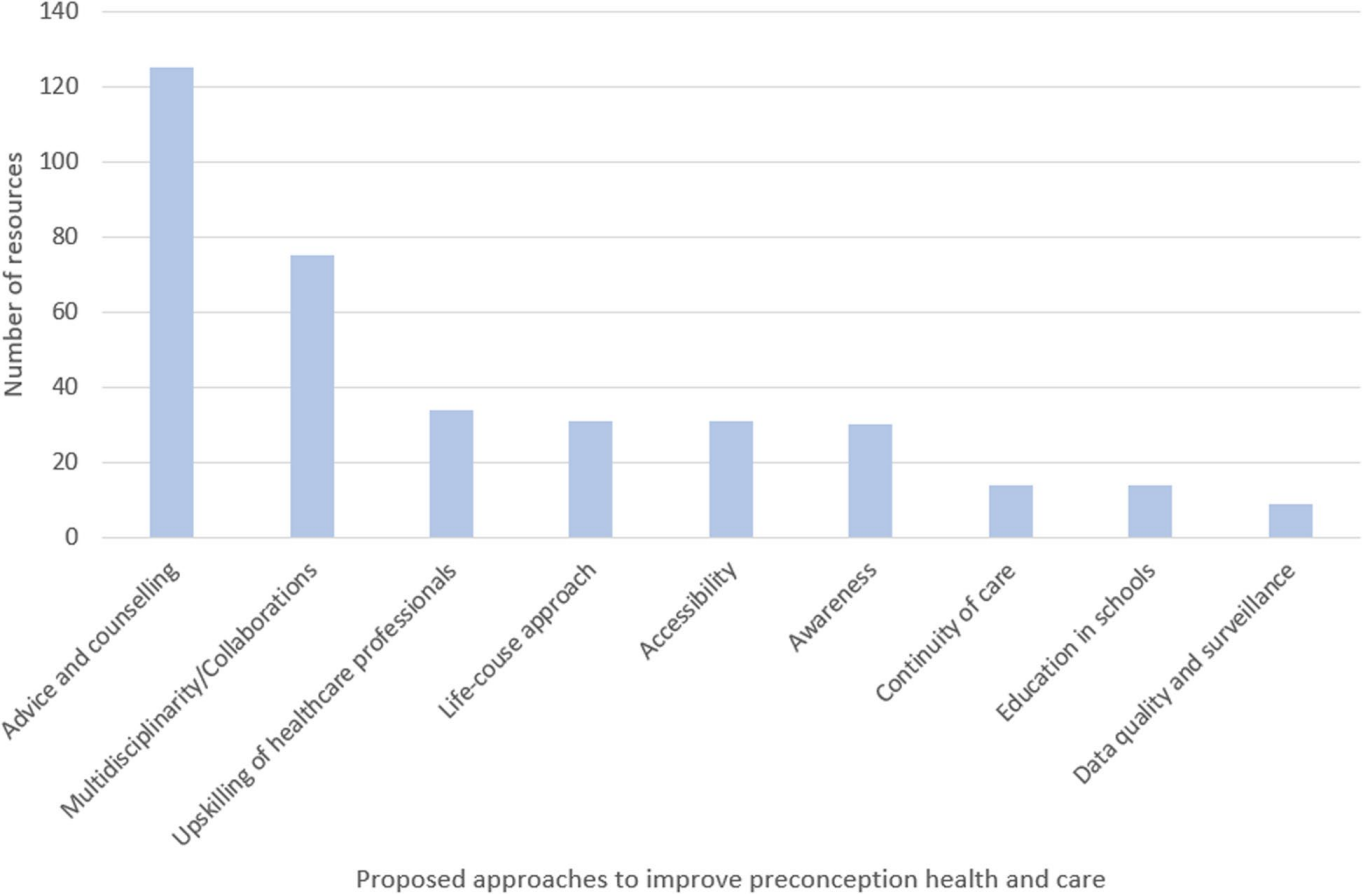
Frequency of reporting of proposed approaches to improve preconception health and care

Resources advocated for interdisciplinary collaborations among professionals (*n* = 75, 27%) (see Additional file 7), alongside efforts to upskill HCPs (*n* = 34, 12.2%) on topics such as mental health, bodyweight-related issues, suitable folic acid supplementation messaging, and identification of domestic abuse [29–35]. Resources also supported the adoption of a life-course approach (*n* = 31, 11.1%) and highlighted the importance of improving access to preconception care (*n* = 31, 11.1%), increasing awareness of preconception health and care (*n* = 30, 10.8%), and maintaining continuity of care where applicable, by involving HCPs such as GPs and specialists to create coordinated healthcare provision across different life stages and settings (*n* = 14, 5%). To increase awareness and understanding of preconception health and care from an early age, schools were particularly highlighted (*n* = 14, 5%). Finally, nine (3.2%) resources recognised the need for improved health surveillance and suggested enhancing data collection processes, including improving the quality and completeness of maternity data recorded at booking appointments. The frequency of each of these approaches is shown in Fig. 3.

#### Preconception health-influencing factors

A wide range of preconception health-influencing factors was addressed in the reviewed resources, including biomedical (e.g., physical health conditions), behavioural (e.g., smoking), and social and environmental factors (e.g., wider determinants of health).

The presence of pre-existing physical health conditions was the most highly cited health-related factor (*n* = 155, 55.8%), and it was often linked to medication use (*n* = 149, 53.6%). Diabetes (*n* = 93) and epilepsy (*n* = 39) were the most frequently mentioned conditions, and sodium valproate (*n* = 23) was the most frequently mentioned medication. Mental health conditions were mentioned less frequently (*n* = 81, 29.1%), although there was a recognition that mental health services require improvement. Folic acid supplementation was detailed in 50% of the included resources (*n* = 139). In particular, the supplementation of 400μg/day was recommended in *n* = 50 resources, and the higher dose of 5mg/day in *n* = 56 resources, including resources that did not solely aim to provide advice for people with increased medical risks. Among the observed behaviours, sleep was mentioned the least frequently (*n* = 3, 1.1%).

Forty-eight (17.3%) resources emphasised the influence of wider determinants on preconception health, including socioeconomic status and ethnicity. Resources proposed that multi-faceted preconception interventions should be designed to prevent and reduce health inequalities, and supported individualised approaches tailored to those experiencing different needs (e.g., based on ethnicity, culture, financial limitations, life circumstances) (e.g., [4, 34, 36, 37]).

**Fig. 4:**
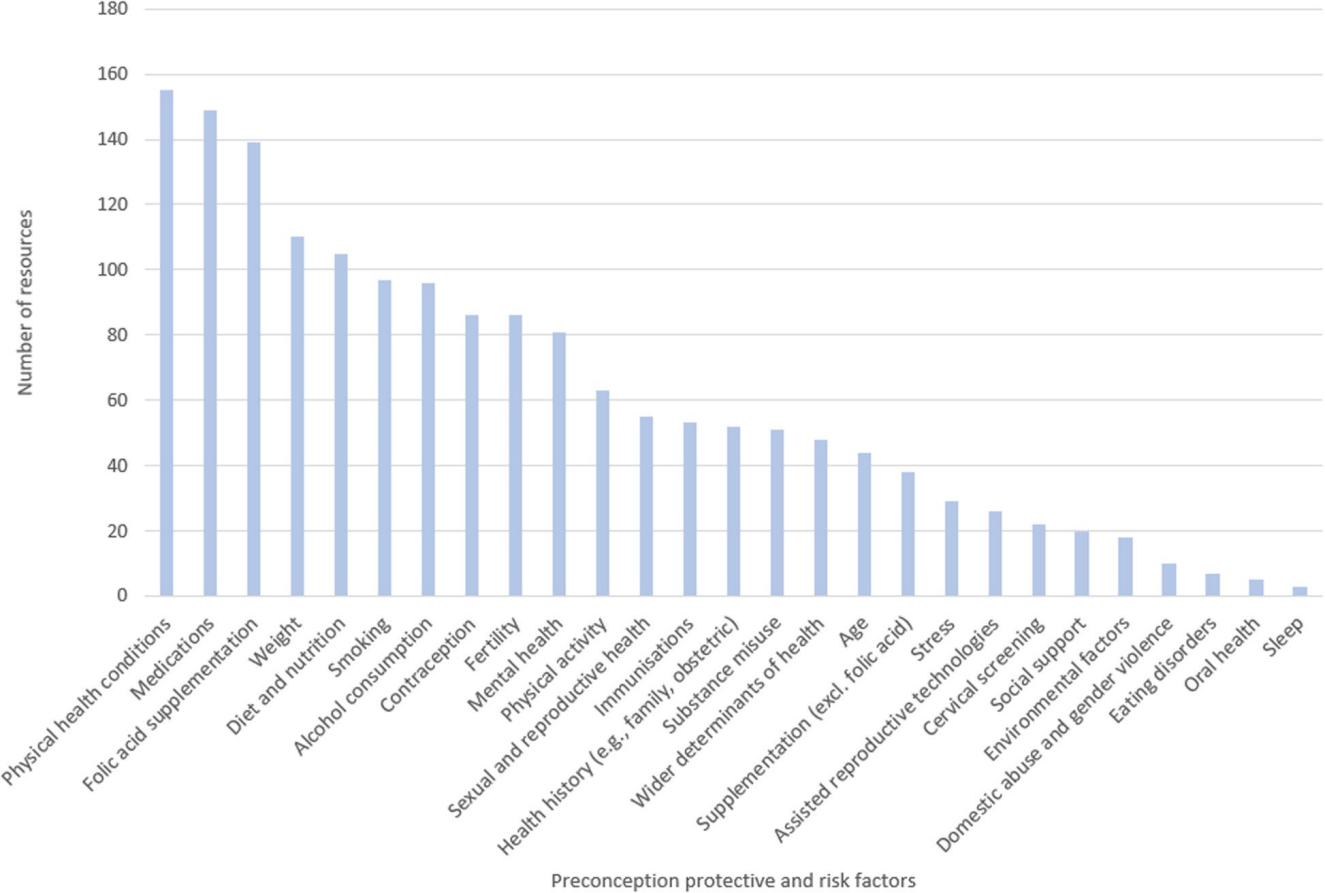
Frequency of reporting of identified preconception health protective and risk factors

All identified preconception health-influencing factors, and the frequency of mentions, are presented in Fig. 4. Excerpts from the three most commonly observed factors can be found in Table 4.

**Table 4:** Excerpts extracted from reviewed resources, pertaining to the three most cited themes

*Themes*	*Excerpts (audience: professionals)*	*Excerpts (audience: public)*
Pre-existing physical conditions	“Women are entering pregnancy with more pre-existing problems, including both significant mental, learning and physical health disorders (including obesity, epilepsy, type 2 diabetes), as well as complex social challenges.” (Public Health England, 2018) “Managing long-term conditions for physical […] health […] reduces risks to health and improves pregnancy outcomes.” (National Institute for Health Research, 2017)	“If you have a long-term condition, such as epilepsy or diabetes, it could affect the decisions you make about your pregnancy […]. Before you get pregnant, have a discussion with your specialist or a GP about getting pregnant.” (NHS, 2020) “Contact your consultant or GP for advice if you have any underlying health conditions for example; epilepsy, diabetes, heart disease, high blood pressure, asthma, autoimmune disorders” (Northern Health and Social Care Trust, 2021)
Medication use	“In order to enable informed decisions and choice, women […] must be given accurate information and counselling about contraception, conception, pregnancy […]. In particular, it is recommended that there should be a discussion with women and girls of childbearing potential (including young girls who are likely to need treatment into their childbearing years), and their parents and/or carers if appropriate, concerning the risk of […] drugs causing malformations and possible neurodevelopmental impairments in an unborn baby.” (Medicines and Healthcare Products Regulatory Agency, 2021)	“Not all medicines are safe to take when you're […] planning a pregnancy, whether they're on prescription or medicines you can buy in a pharmacy or shop If you take prescribed medicine and you're planning to get pregnant, talk to a doctor Do not stop taking your medicine without talking to a doctor.” (NHS, 2020)
Folic acid supplementation	“Advise women with diabetes who are planning a pregnancy to take folic acid (5 mg/day) until 12 weeks of gestation to reduce the risk of having a baby with a neural tube defect.” (NICE, 2015)	“If you have diabetes and are trying to get pregnant, you should take 5 mg (mg) of folic acid each day (and until you are 12 weeks pregnant). A doctor will have to prescribe this, because you cannot buy 5mg tablets from a pharmacy or shop without a prescription.” (NHS, 2021)

Advice provided regarding preconception health-influencing factors was often accompanied by information concerning associated adverse maternal and infant outcomes. For example, when discussing pregnancies among young individuals (e.g., under 20 years of age), resources often covered the description of potential risks, including stillbirth, infant mortality and poor maternal mental health, as well as associated risk factors such as smoking and unplanned pregnancies (e.g., [4, 38, 39]). For most of the behaviours, advice included having conversations with HCPs, who were encouraged to avail of referral pathways to ensure adequate support. The included resources recommended that healthcare services should incorporate discussions on preconception health behaviours as part of routine practice [40, 41]. However, resources addressing preconception health-influencing factors also highlighted that available support is currently unsatisfactory and access to care is inconsistent in many areas (e.g., smoking cessation, fertility services) [40, 42]. This may be attributed to the fragmented design and delivery of services [40]. In this review, inconsistencies were found in relation to some of the advice provided, for example regarding safe preconception alcohol intakes, recommended physical activity levels, and dietary habits including caffeine consumption. For instance, while certain resources suggested that caffeine should be limited to 200mg/day, others proposed there is no robust evidence to support such recommendations [43–48].

### Gaps in knowledge (Research question 4)

Certain knowledge gaps were emphasised in the resources reviewed. For example, resources highlighted the need to further investigate the most suitable preconception care delivery methods and interventions, and particularly mentioned interventions aiming to promote physical and mental health, lower levels of alcohol consumption before pregnancy for both men and women, and improve the uptake of folic acid supplementation [49, 50]. The resources also recognised the need to further investigate topics such as the predictors of preconception care engagement and to advance the current understanding of preconception health-influencing factors and related health conditions (e.g., [22, 46, 51]). Although many resources acknowledged the influence that wider determinants can have on preconception health, resources also highlighted that further efforts are required to adequately address the major disparities that persist (e.g., in relation to ethnicity and socioeconomic status) [26, 27]. Suggestions included the development of suitable preconception weight measurement methods for minority ethnic groups, and tailored ways to promote the uptake of folic acid and other supplements among disadvantaged groups [34, 50].

### Audit – Northern Ireland (Research question 5)

**Fig. 5:**
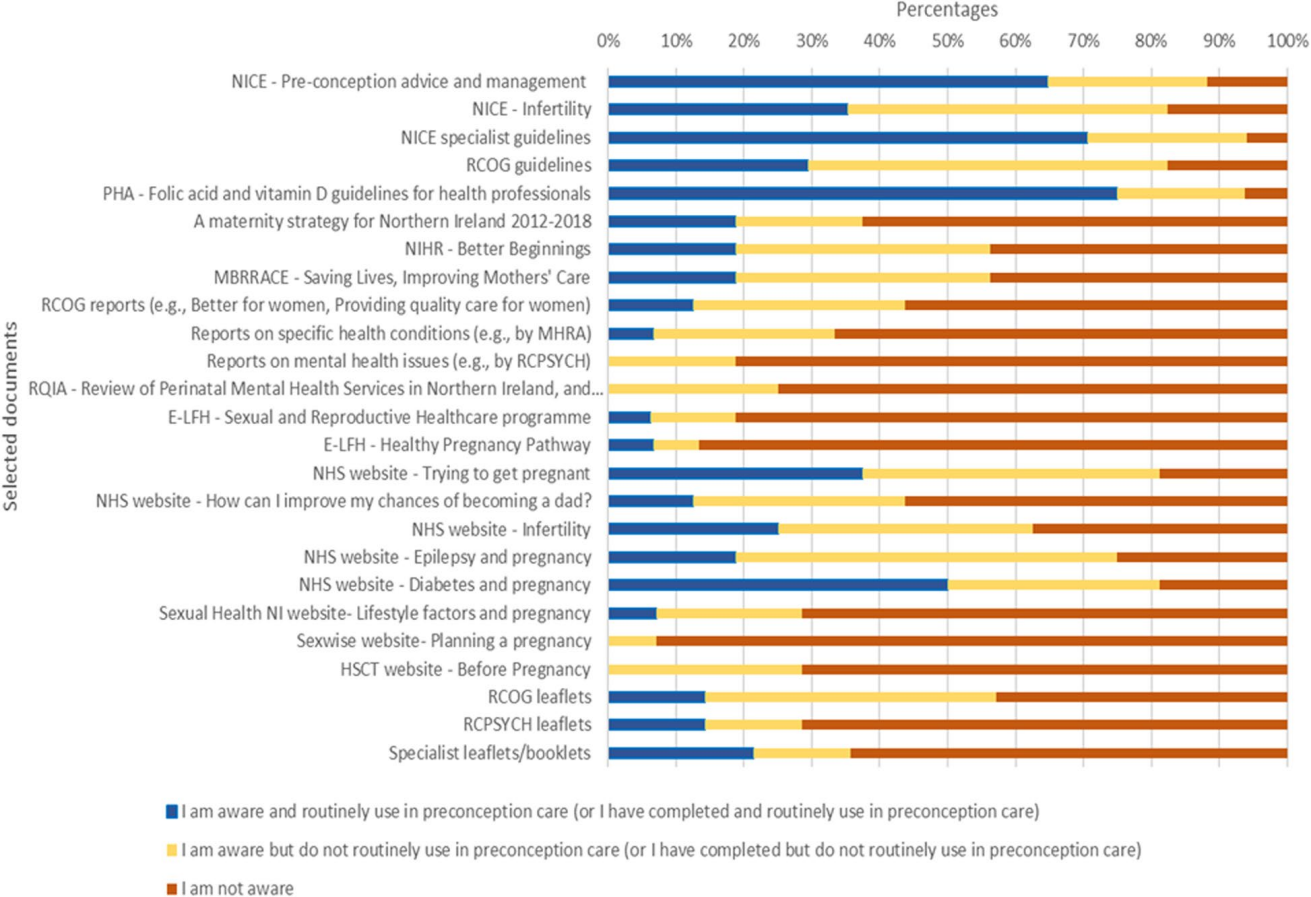
Self-reported awareness and use of selected reviewed resources among audited healthcare professionals (*n* = 17). Abbreviations: NICE: National Institute for Health & Clinical Excellence; RCOG: Royal College of Obstetricians & Gynaecologists; PHA: Public Health Agency; NIHR: National Institute for Health and Care Research; MBRRACE: Mothers and Babies – Reducing Risk through Audits and Confidential Enquiries across the UK; MHRA: Medicines and Healthcare products Regulatory Agency; RCPSYCH: Royal College of Psychiatrists; RQIA: Regulation and Quality Improvement Authority; E-LFH: E-Learning For Healthcare; NHS: Nation Health Service; NI: Northern Ireland; HSCT: Health and Social Care Trust

Seventeen HCPs in Northern Ireland completed the audit checklist, including five (diabetes) specialists, five general practitioners (GPs) and a GP trainee, three midwives, two pharmacists, and an endocrinologist. Fifteen participants were aware of the NICE Clinical Knowledge Summary on preconception advice and management [21], with the majority (*n* = 11) reporting its routine use in preconception care. Of the specialist NICE resources, the one focusing on the management of diabetes from the preconception to the postpartum period [52] was most often recognised. Most participants were aware of guidelines from the Royal College of Obstetricians and Gynaecologists, and specifically mentioned guidelines focusing on obesity, epilepsy, diabetes and mental health issues (e.g., [53, 54]). Another resource most participants (*n* = 15) were aware of and routinely used in preconception care was a brief guideline by the Northern Ireland Public Health Agency, a body responsible for providing health protection and improvement in Northern Ireland, on the topic of folic acid and vitamin D [55]. Awareness of relevant preconception health and care policies, strategies, reports and e-learning resources was not notably prevalent. Similarly, the use and awareness of leaflets and web pages directed at the public was generally poor, except for the NHS web page titled ‘Trying to get pregnant’ [56] (*n* = 13), and those addressing the management of diabetes when planning pregnancies (e.g., [57, 58]). The frequency of participants’ self-reported awareness and use of selected reviewed resources is presented in Fig. 5.

The audit identified two additional resources that were not identified in the searches [59, 60]. One participant also mentioned using the Mum and Baby Academy website, a UK-based online provider of academically-approved free courses for professionals, for continual professional development [61]. This website was searched, and three additional e-learning courses were included in the review [62–64].

### Patient and public involvement and engagement

Early engagement with eight PPIE representatives during two workshops indicated their belief that preconception health is an important aspect of health, thereby helping with the conceptualisation of the review and priority setting. Subsequently, two PPIE representatives (DT, TS) directly collaborated on the review protocol [12], and provided feedback on the interpretation of the results. Overall, they expressed an interest in the topics under review by asking about health-influencing factors the public may not be familiar with (e.g., pelvic floor health, artificial reproductive technologies), suggesting clarifications on certain concepts and mechanisms (e.g., the practical implementation of a life-course approach, the specific strategies that can raise the public’s awareness of preconception health and care), and inquiring about knowledge gaps (e.g., the reasons why access to care is lacking). A further workshop was held with three other PPIE representatives to design a summary infographic (Fig. 2). In the discussions that unfolded during this workshop, representatives suggested that many individuals seek preconception health-related advice only if experiencing difficulties conceiving, and highlighted the importance of increasing preconception health and care awareness from an early age.

## Discussion

### Overview of resources identified

This scoping review identified a large number of policies, strategies, guidelines, frameworks and recommendations in the UK and Ireland addressing topics related to preconception health and care (*n* = 278). These resources recognised the period preceding conception as an opportunity to optimise preconception health, by identifying common approaches for preconception health and care optimisation and a wide range of preconception health-influencing factors. Preparation for pregnancy was emphasised for women more than for men or same sex partners, and especially those with pre-existing physical and mental health conditions. Accessing women’s health services was recognised as challenging and fear-inducing for people with pre-existing conditions planning a pregnancy [65], which can result in poorer access to care, unplanned pregnancies or avoidance of pregnancy altogether [40].

In this review, a wide variety of resource types were identified for healthcare professionals and the public, including, for example, policies, strategies, action plans, e-learning resources, reports, guidelines, leaflets, and booklets. However, the preconception period was often not their primary or sole topic. This finding is supported by a previous review on the availability and quality of guidelines for preconception care, which identified numerous guidelines referring to preconception but only 11 guidelines primarily focusing on preconception care [11]. Many websites with recommendations and guidance were found, which may be considered promising as digital platforms can have a wider reach, promote behaviour change, and be suitable for educational purposes at scale [66, 67]. Online engagement is especially opportune when resources are easy to access, reputable, evidence-based and, where applicable, include advice from peers with similar experiences [66] or interactive elements. Mobile phone apps may also support the optimisation of preconception health, although the evidence is limited [68]. Other public-facing resources identified were booklets and leaflets which provided useful information, but seeking relevant professional advice before taking or avoiding actions based on the information relayed in these materials was often encouraged.

The findings of the review showed that, since 2015, the availability of resources has increased, perhaps suggesting increased awareness of the benefits of preconception health and care. However, the establishment of a strong culture of preconception care in the UK and Ireland still necessitates dedicated efforts and co-produced interventions.

### Main themes identified

Nine different approaches were identified for improving preconception health and care. However, many of these rely on the role of HCPs and health services, thus reflecting the scope of many resources that was mainly confined to clinical rather than public health settings. Similar to other pressing healthcare priorities, the progression towards the establishment of a culture of preconception care necessitates a significant allocation of resources. One of the barriers to provision of preconception care is the limited capacity of healthcare systems and other networks to provide timely and accessible preconception care. Unrealistic expectations placed solely upon HCPs who are working within an already pressurised healthcare system should be avoided. Other awareness-raising opportunities outside the healthcare system were mentioned, including schools and (digital) media campaigns. Overall, a multi-level approach to preconception care combining bottom-up mobilisation of individuals and communities with public health top-down initiatives from governmental bodies was encouraged [34, 36], with particular support for initiatives designed with stakeholders (including the public).

This review also identified many modifiable and non-modifiable preconception health-influencing factors, placing stronger emphasis on clinical characteristics such as pre-existing physical health conditions, and to a lesser extent mental health conditions. However, there was a recognition that preconception health risk and protective factors do not occur in isolation but are interconnected and often clustered, with pregnancies not being conceived in isolation from “the ocean of socioeconomic, cultural, family, corporate, governmental/political forces surrounding it” [69]. Certain inconsistencies were identified concerning physical activity, caffeine consumption and alcohol intake in both men and women, indicating a need for clearer and more consistent advice (although variations may also be influenced by changes in recommendations over time). Efforts should be made to remove contrasting and potentially outdated advice, especially in online resources.

### Building on previous research

The present review aimed to build upon a previous review of preconception care policy, guidelines, recommendations and services [3]. The previous review identified a focus on recommendations for women with chronic diseases and, while the current review found a substantial number of resources available for all women, pre-existing physical health conditions were frequently mentioned. Both reviews found a limited focus on men, identified heterogeneous advice, and overall highlighted a need for the development of evidence-based resources for preconception health and care, to accompany a clear strategy for health promotion across the childbearing population [3]. However, there are also differences between the two reviews. One way to illustrate these differences is through the varied guidance provided regarding alcohol consumption. The advice presented pertaining to the UK from the earlier review indicated that women should limit their alcohol intake to 1–2 units once or twice a week, while the advice from the current review exhibited great variability, with abstinence also being suggested. Additionally, the present review encompassed information regarding the fortification of flour with folic acid, a topic that was still under consideration in the UK during the publication of the prior review [3].

### Further research

This review identified gaps in knowledge, offering recommendations for potential future research. For example, further efforts should aim to investigate preconception care engagement, preconception health-related factors and conditions, delivery of non-conflicting advice, and suitable interventions (e.g., to promote physical and mental health) [22, 49, 70]. This may help clarify the inconsistencies found in the advice provided. While many approaches that can be used to improve preconception health and care were identified in this review, further research needs to be undertaken to explore clear and innovative preconception health-promotion means, and address the needs of diverse populations at different life stages across the reproductive life span [70]. Although many resources acknowledged the influence wider determinants can have on preconception health, research is still needed to further investigate how to suitably address the ongoing disparities in relation to ethnicity, socioeconomic status and further inequalities (e.g., educational attainment) [26, 27]. Overall, findings from this review indicate that further efforts are required to foster and support the development of a culture of preconception care, also including men and partners when applicable.

### Audit – Northern Ireland

Auditing HCPs’ use and awareness of preconception care resources in Northern Ireland contextualised the review findings, offering insights on the reach of identified resources. The audit led to the identification of five additional resources, reinforcing the comprehensiveness of the review. Most participants were aware of the NICE Clinical Knowledge Summary resource on preconception advice and management [21], and there was repeated recognition and use of resources on diabetes management, although this finding may be influenced by the inclusion of specialist diabetes nurses. Overall, the audit supported the results of this review, reinforcing the need for further upskilling of HPCs on topics related to preconception health and care (e.g., mental health, paternal factors). Overwhelming HCPs with new resources or solely relying on them may, however, not be desirable. While referral pathways for specialist cases may be used, the results of the review suggested that there is still need for improvement in how these are implemented. However, this may be limited by resource allocation within the healthcare system.

### Patient and public involvement and engagement

Representatives from the PPIE panel were invited to co-produce this scoping review. There was a level of interest shown towards preconception health and care, as representatives believed preconception health to be an important aspect of health and wanted to deepen their knowledge on the evidence available. They asked for further clarifications on certain aspects of preconception health and mentioned the importance to increase awareness from an early age. Overall, PPIE strategies suggested that the public may be receptive to preconception health-related messaging, if presented in a clear yet thorough way, and emphasised the importance of tailoring the delivery of advice to align with the interests and concerns of the public.

### Strengths and limitations

This scoping review was conducted to explore the breadth of grey literature on preconception health and care populating the scene for over a decade in the UK and Ireland. A systematic approach was followed, thus locating, synthesising and presenting evidence from a broad and diverse topic in a clear manner, accessible to stakeholders [14]. The addition of the audit undertaken to explore preconception care in Northern Ireland with 17 HCPs represents a further strength, as well as the contribution and input from PPIE representatives. The panel included adults between 18 and 45 years old, and engaging with younger and older people with reproductive potential may provide additional insights on their desires and interests.

Because the study was intended as a broad scoping review, it included a heterogenous group of resources. Resources explicitly addressing only the interconception period were excluded, although it is recognised as an important time during which individuals are likely to engage with health-promoting messages and be in frequent contact with HCPs [71]. Regardless of the measures taken to limit missing relevant citations, potentially eligible resources may have been missed due to the limited number of databases searched, the language restriction applied to the searches, the narrowed timeline and the search terms used. Additionally, relevant resources may have been missed if not available online. However, this limitation was minimised by the inclusion of the audit in Northern Ireland, which allowed the identification of five additional resources. By including Google Advanced Search, the replicability of the searches is limited due to the algorithm governing these types of searches [72]. Moreover, the quality of the advice provided in the public-facing resources included in this review was not assessed. Finally, because this review focused only on the evidence available in the UK and Ireland, findings may not be generalisable to other countries.

## Conclusion

This scoping review identified and analysed preconception health-related policies, strategies, guidelines, frameworks, and recommendations in the UK and Ireland (*n* = 278). It identified several approaches to optimise preconception care delivery, mainly in resources directed at a professional audience, as well as preconception health-influencing factors, of which pre-existing health conditions were the most frequently mentioned. A specialised audit contextualised the findings relevant to Northern Ireland by highlighting the use and awareness of identified resources among HCPs.

The reviewed resources advocated for both better individual support and structural improvements to optimise health outcomes, and identified the need to further investigate preconception health-related factors. While more research is still needed (e.g., to address the inconsistencies in preconception health messages, tackle inequalities, support the widespread implementation of guidelines), this review provides an overview of resources available to HCPs and the public that can be used to optimise preconception health and care.

### Supplementary Information

Supplementary Material 1.

## Data Availability

Data is provided within the manuscript or supplementary information files.

## Abbreviations

NICE: National Institute for Health & Clinical Excellence
RCOG: Royal College of Obstetricians & Gynaecologists
PHA: Public Health Agency
NIHR: National Institute for Health and Care Research
MBRRACE: Mothers and Babies – Reducing Risk through Audits and Confidential Enquiries across the UK
MHRA: Medicines and Healthcare products Regulatory Agency
RCPSYCH: Royal College of Psychiatrists
RQIA: Regulation and Quality Improvement Authority
E-LFH: E-Learning For Healthcare
NHS: Nation Health Service
NI: Northern Ireland
HSCT: Health and Social Care Trust

## References

[1] World Health Organization. Women of reproductive age (15-49 years) population (thousands). No date. https://www.who.int/data/gho/indicator-metadata-registry/imr-details/women-of-reproductive-age-(15-49-years)-population-(thousands). Accessed 29 May 2023.

[2] World Health Organization. Policy brief: preconception care – maximising the gains for maternal and child health. 2013. https://www.who.int/publications/i/item/WHO-FWC-MCA-13.02. Accessed 22 Apr 2022.

[3] Shawe J, Delbaere I, Ekstrand M (2015). Preconception care policy, guidelines, recommendations and services across six European countries: Belgium (Flanders), Denmark, Italy, the Netherlands, Sweden and the United Kingdom. Eur J Contracept Reprod Health Care.

[4] Public Health England. Making the case for preconception care. 2018. https://www.gov.uk/government/publications/preconception-care-making-the-case. Accessed 5 Dec 2022.

[5] Broussard DL, Sappenfield WB, Fussman C (2011). Core state preconception health indicators: a voluntary, multi-state selection process. Matern Child Health J.

[6] Schoenaker DAJM, Stephenson J, Connolly A (2021). Characterising and monitoring preconception health in England: a review of national population-level indicators and core data sources. J Dev Orig Health Dis.

[7] Schoenaker DAJM, Stephenson J, Smith H (2023). Women's preconception health in England: a report card based on cross-sectional analysis of national maternity services data from 2018/2019. BJOG.

[8] Evans H, Buck D. Tackling multiple unhealthy risk factors. 2018. https://www.kingsfund.org.uk/sites/default/files/2018-03/Tackling%20multiple%20unhealthy%20risk%20factors%20-%20full%20report.pdf. Accessed 5 Dec 2022.

[9] Fleming TP, Watkins AJ, Velazquez MA (2018). Origins of lifetime health around the time of conception: causes and consequences. Lancet.

[10] Stephenson J, Heslehurst N, Hall J (2018). Before the beginning: nutrition and lifestyle in the preconception period and its importance for future health. Lancet.

[11] Dorney E, Boyle JA, Walker R (2022). A systematic review of clinical guidelines for preconception care. Semin Reprod Med.

[12] Cassinelli EH, McKinley MC, Kent L (2023). Preconception health and care policies, strategies and guidelines in the UK and Ireland: a scoping review protocol. BMJ Open.

[13] Peters MDJ, Marnie C, Tricco AC (2020). Updated methodological guidance for the conduct of scoping reviews. JBI Evid Synth.

[14] Arksey H, O'Malley L (2005). Scoping studies: towards a methodological framework. Int J Soc Res Methodol.

[15] Tricco AC, Lillie E, Zarin W (2018). PRISMA extension for scoping reviews (PRISMA-ScR): Checklist and Explanation. Ann Intern Med.

[16] Godin K, Stapleton J, Kirkpatrick SI (2015). Applying systematic review search methods to the grey literature: a case study examining guidelines for school-based breakfast programs in Canada. Syst Rev.

[17] Canadian Institutes of Health Research. Strategy for Patient-Oriented Research. Capacity development framework. 2015. https://cdn.dal.ca/content/dam/dalhousie/pdf/research-services/REB/Newsletter/spor_capacity_development_framework-en.pdf. Accessed 26 May 2022.

[18] National Institute for Health and Care Research. Payment guidance for researchers and professionals. 2022. https://www.nihr.ac.uk/documents/payment-guidance-for-researchers-and-professionals/27392. Accessed 30 Jul 2023.

[19] Staniszewska S, Brett J, Simera I (2017). GRIPP2 reporting checklists: tools to improve reporting of patient and public involvement in research. Res Involv Engagem.

[20] National Institute for Health & Clinical Excellence. Postnatal care. 2021. https://www.nice.org.uk/guidance/ng194. Accessed 16 Nov 2023.

[21] National Institute for Health & Clinical Excellence. Pre-conception - advice and management (Clinical Knowledge Summaries). 2022. https://cks.nice.org.uk/topics/pre-conception-advice-management/. Accessed 15 Dec 2022.

[22] British Medical Association. Alcohol and Pregnancy. Preventing and managing fetal alcohol spectrum disorders. 2007. Updated in 2016. https://www.bma.org.uk/media/2082/fetal-alcohol-spectrum-disorders-report-feb2016.pdf. Accessed 12 Dec 2022.

[23] Sher J. Prepared for Pregnancy? Preconception health, education and care in Scotland. 2016. https://www.stor.scot.nhs.uk/bitstream/handle/11289/578820/prepared-for-pregnancy-j-sher-may-2016.pdf?sequence=1&isAllowed=y. Accessed 5 Dec 2022.

[24] NI Direct. Infertility. 2018. Updated in 2023. https://www.nidirect.gov.uk/conditions/infertility. Accessed 10 Feb 2023.

[25] Public Health England. Maternity high impact area: reducing the inequality of outcomes for women from Black, Asian and Minority Ethnic (BAME) communities and their babies. 2020. https://assets.publishing.service.gov.uk/government/uploads/system/uploads/attachment_data/file/942480/Maternity_high_impact_area_6_Reducing_the_inequality_of_outcomes_for_women_from_Black__Asian_and_Minority_Ethnic__BAME__communities_and_their_babies.pdf. Accessed 8 Dec 2022.

[26] National Institute for Health & Clinical Excellence. Antenatal and postnatal mental health: clinical management and service guidance. 2014. Updated in 2020. https://www.nice.org.uk/guidance/cg192/resources/antenatal-and-postnatal-mental-health-clinical-management-and-service-guidance-pdf-35109869806789. Accessed 12 Dec 2022.

[27] National Institute for Health & Clinical Excellence. Sapropterin for treating hyperphenylalaninaemia in phenylketonuria. 2022. https://www.nice.org.uk/guidance/ta729/resources/sapropterin-for-treating-hyperphenylalaninaemia-in-phenylketonuria-pdf-82611202437061. Accessed 12 Dec 2022. 40789119

[28] Department of Health, Social Services and Public Safety. A strategy for maternity care in Northern Ireland 2012-2018. 2012. https://www.health-ni.gov.uk/sites/default/files/publications/dhssps/maternitystrategy.pdf. Accessed 5 Dec 2022.

[29] National Institute for Health & Clinical Excellence. Maternal and child nutrition (PH11). 2008. Updated in 2014. https://www.nice.org.uk/guidance/ph11/resources/maternal-and-child-nutrition-pdf-1996171502533. Accessed 12 Dec 2022.

[30] Safefood. The folate status of pregnant women in the Republic of Ireland; the current position. 2017. https://www.hse.ie/eng/about/who/acute-hospitals-division/woman-infants/national-reports-on-womens-health/folate-status-in-pregnant-women-in-republic-of-ireland.pdf. Accessed 12 Dec 2022.

[31] Public Health England. Maternity high impact area: Supporting good parental mental health. 2020. https://assets.publishing.service.gov.uk/government/uploads/system/uploads/attachment_data/file/942475/Maternity_high_impact_area_2_Supporting_good_parental_mental_health.pdf. Accessed 06 Dec 2022.

[32] Health Service Executive IE. Specialist perinatal mental health services - model of care for Ireland. 2017. https://www.hse.ie/eng/services/list/4/mental-health-services/specialist-perinatal-mental-health/specialist-perinatal-mental-health-services-model-of-care-2017.pdf. Accessed 17 Jan 2023.

[33] Children’s Alliance. The health and wellbeing of children in the early years. 2021. https://www.ecsdn.org/wp-content/uploads/2021/11/WG1-EarlyYears-Oct2021.pdf. Accessed 12 Dec 2022.

[34] All Party Parliamentary Group. Maternal obesity - a report by the All-Party Parliamentary Group on a fit and healthy childhood. 2017. https://fhcappg.org.uk/wp-content/uploads/2018/04/moreportfinal_june2017.pdf. Accessed 6 Dec 2022.

[35] eLearning for healthcare. Healthy pregnancy pathway. No date. https://e-lfh.org.uk/healthy-pregnancy-pathway/index.html. Accessed 7 Dec 2022.

[36] Public Health England. Maternity high impact area: Improving planning and preparation for pregnancy. 2020. https://assets.publishing.service.gov.uk/government/uploads/system/uploads/attachment_data/file/942474/Maternity_high_impact_area_1_Improving_planning_and_preparation_for_pregnancy.pdf. Accessed 7 Dec 2022.

[37] MacAskill L, MacDonald V, Kellock J et al. Maternal and child nutrition best practice guidance. 2011. http://www.forhighlandschildren.org/4-icspublication/index_6_3071689108.pdf. Accessed 15 Dec 2022.

[38] Royal College of Obstetricians and Gynaecologists. Better for women - improving the health and wellbeing of girls and women. 2019. https://www.rcog.org.uk/media/h3smwohw/better-for-women-full-report.pdf. Accessed 5 Dec 2022.

[39] DG Health and Wellbeing. Preconception health toolkit. 2016. https://sexualhealthdg.co.uk/downloads/Preconception%20Health%20Toolkit.pdf. Accessed 7 Dec 2022.

[40] Public Health England. Health of women before and during pregnancy: health behaviours, risk factors and inequalities. 2019. https://allcatsrgrey.org.uk/wp/download/obstetrics/Health_of_women_before_and_during_pregnancy_2019.pdf. Accessed 5 Dec 2022.

[41] UK Health Security Agency. Health matters: reproductive health and pregnancy planning. 2018. https://ukhsa.blog.gov.uk/2018/06/26/health-matters-reproductive-health-and-pregnancy-planning/. Accessed 15 Dec 2022.

[42] Faculty of Sexual and Reproductive Healthcare, Royal College of Obstetricians and Gynaecologists, British Association for Sexual Health. Promoting Scotland’s sexual and reproductive health: a joint manifesto. 2021. https://www.fsrh.org/documents/joint-manifesto-scotland-elections-sexual-reproductive-health/. Accessed 3 Nov 2022.

[43] Royal College of Obstetricians and Gynaecologists, Royal College of Midwives. Healthy eating and vitamin supplements in pregnancy patient information leaflet. 2022. https://www.rcog.org.uk/media/nkvpl2mn/healthy-eating-vitamin-supplements-pregnancy-patient-information.pdf. Accessed 6 Jan 2023.

[44] Tommy’s. Fertility treatment options. 2021. https://www.tommys.org/pregnancy-information/planning-a-pregnancy/fertility-and-causes-of-infertility/fertility-treatment-options. Accessed 6 Jan 2023.

[45] National Institute for Health & Clinical Excellence. Fertility problems: assessment and treatment (CG156). 2013. Updated in 2017. https://www.nice.org.uk/guidance/cg156/resources/fertility-problems-assessment-and-treatment-pdf-35109634660549. Accessed 14 Nov 2023.

[46] National Institute for Health & Clinical Excellence. Infertility (clinical knowledge summaries). 2018. https://cks.nice.org.uk/topics/infertility/. Accessed 15 Dec 2022.

[47] Tommy’s. How to improve male fertility. 2021. https://www.tommys.org/pregnancy-information/planning-a-pregnancy/are-you-ready-to-conceive/how-improve-male-fertility. Accessed 14 Nov 2023.

[48] NI Direct. Infertility. 2018. Updated 2023. https://www.nidirect.gov.uk/conditions/infertility. Accessed 10 Feb 2023.

[49] Department of Health. Annual Report of the Chief Medical Officer, 2014. 2015. https://assets.publishing.service.gov.uk/government/uploads/system/uploads/attachment_data/file/595439/CMO_annual_report_2014.pdf. Accessed 5 Dec 2022.

[50] National Institute for Health Research - Dissemination Centre. Better beginnings: improving health for pregnancy. 2017. https://evidence.nihr.ac.uk/wp-content/uploads/2020/03/Better-beginnings-web-interactive.pdf. Accessed 6 Dec 2022.

[51] EUROCAT. Primary prevention of congenital anomalies. 2012. https://eu-rd-platform.jrc.ec.europa.eu/sites/default/files/EUROCAT-EUROPLAN-Primary-Preventions-Reccomendations.pdf. Accessed 15 Dec 2022.

[52] Denison FC, Aedla NR, Keag O, et al. on behalf of the Royal College of Obstetricians and Gynaecologists. Care of women with obesity in pregnancy green-top guideline no. 72. 2018. https://obgyn.onlinelibrary.wiley.com/doi/epdf/10.1111/1471-0528.15386. Accessed 5 Jan 2023. 10.1111/1471-0528.15386 30465332

[53] National Institute for Health & Clinical Excellence. Diabetes in pregnancy: management from preconception to the postnatal period. 2015. Updated in 2020. https://www.nice.org.uk/guidance/ng3. Accessed 18 Aug 2023. 32212588

[54] Royal College of Obstetricians and Gynaecologists. Management of women with mental health issues during pregnancy and the postnatal period (Good Practice No.14). 2011. https://www.rcog.org.uk/media/4gikqggv/managementwomenmentalhealthgoodpractice14.pdf. Accessed 9 Jan 2023.

[55] Public Health Agency, Health and Social Care Board. Folic acid and vitamin D guidelines for health professionals. 2017. https://www.publichealth.hscni.net/sites/default/files/FOLIC%20ACID%20and%20VITAMIN%20D%20Guidelines%202017.pdf. Accessed 6 Jan 2023.

[56] NHS. Trying to get pregnant. 2020. https://www.nhs.uk/pregnancy/trying-for-a-baby/trying-to-get-pregnant/ Accessed 16 Jun 2023.

[57] Diabetes UK. Planning for a pregnancy when you have diabetes. 2018. Updated 2022. https://www.diabetes.org.uk/guide-to-diabetes/life-with-diabetes/pregnancy. Accessed 15 Dec 2022.

[58] Diabetes UK. Preconception care for women with diabetes. 2015. https://www.diabetes.org.uk/professionals/position-statements-reports/specialist-care-for-children-and-adults-and-complications/preconception-care-for-women-with-diabetes. Accessed 16 Jun 2023.

[59] South Eastern Health and Social Care Trust. Maternity. 2022. https://setrust.hscni.net/service/maternity-2/. Accessed 1 Jun 2023.

[60] Diabetes UK. Evidence-based nutrition guidelines for the prevention and management of diabetes. 2018. https://diabetes-resources-production.s3.eu-west-1.amazonaws.com/resources-s3/2018-03/1373_Nutrition%20guidelines_0.pdf. Accessed 2 Jul 2023.

[61] Mum and Baby Academy. About the Mum & Baby Academy. No date. https://www.healthprofessionalacademy.co.uk/mum-and-baby/about. Accessed 17 Nov 2023.

[62] Mum and Baby Academy. Pregnancy preconception and planning. No date. https://www.healthprofessionalacademy.co.uk/mum-and-baby/learn/pregnancy-planning-cpd. Accessed 09 Oct 2023.

[63] Mum and Baby Academy. Nutrition in Pregnancy. No date. https://www.healthprofessionalacademy.co.uk/mum-and-baby/learn/nutrition-in-pregnancy-cpd. Accessed 09 Oct 2023.

[64] Mum and Baby Academy. Nutritional supplements in pregnancy. No date. https://www.healthprofessionalacademy.co.uk/mum-and-baby/learn/nutritional-supplements-in-pregnancy-mba. Accessed 9 Oct 2023.

[65] NHS, Tommy’s, King’s College London. Delivering preconception care to women of childbearing age with serious mental illness. 2020. https://www.tommys.org/sites/default/files/legacy/Pre-conception%20care%20and%20serious%20mental%20illness%20V7%20DRAFT.pdf. Accessed 12 Dec 2022.

[66] Walker RE, Quong S, Olivier P (2022). Understanding Preconception Women's Needs and Preferences for Digital Health Resources: Qualitative Study. JMIR Form Res.

[67] Musgrave L, Homer C, Gordon A (2023). Knowledge, attitudes and behaviours surrounding preconception and pregnancy health: an Australian cross-sectional survey. BMJ Open.

[68] Musgrave L, Cheney K, Dorney E (2023). Addressing preconception behavior change through mobile phone apps: Systematic review and meta-analysis. J Med Internet Res.

[69] Sher J. Missed Periods - Scotland’s opportunities for better pregnancies, healthier parents and thriving babies the first time ... and every time. 2016. https://www.basw.co.uk/system/files/resources/basw_74540-4_0.pdf. Accessed 12 Jan 2023.

[70] Boyle JA, Dodd J, Gordon A (2022). Policies and healthcare to support preconception planning and weight management: optimising long-term health for women and children. Public Health Res Pract.

[71] Watson D, Jacob CM, Giles G (2022). A scoping review of nutritional interventions and policy guidelines in the interconception period for prevention of noncommunicable diseases. RFCH.

[72] Briscoe S. Web searching for systematic reviews: a case study of reporting standards in the UK health technology assessment programme. BMC Res Notes. 2015;2016. Erratum. In: BMC Res Notes. 10.1186/s13104-015-1079-y. 10.1186/s13104-015-1079-y PMC4406036 25889619

