# Supplementary material for: Preconception health and care policies, strategies and guidelines in the UK and Ireland: a scoping review
Source: BMC Public Health. 2024 Jun 22;24:1662. doi: 10.1186/s12889-024-19188-0 (PMC11193169; doi:10.1186/s12889-024-19188-0)
Supplement: Supplementary file 1 — Supplementary Material 1. [file 12889_2024_19188_MOESM1_ESM.docx]

# Additional file 1

**Preferred Reporting Items for Systematic reviews and Meta-Analyses extension for Scoping Reviews (PRISMA-ScR) Checklist**

| **SECTION** | **ITEM** | **PRISMA-ScR CHECKLIST ITEM** | **REPORTED ON PAGE #** |
| --- | --- | --- | --- |
| **TITLE** | | | |
| Title | 1 | Identify the report as a scoping review. | 1 |
| **ABSTRACT** | | | |
| Structured summary | 2 | Provide a structured summary that includes (as applicable): background, objectives, eligibility criteria, sources of evidence, charting methods, results, and conclusions that relate to the review questions and objectives. | 2 |
| **INTRODUCTION** | | | |
| Rationale | 3 | Describe the rationale for the review in the context of what is already known. Explain why the review questions/objectives lend themselves to a scoping review approach. | 3-4 |
| Objectives | 4 | Provide an explicit statement of the questions and objectives being addressed with reference to their key elements (e.g., population or participants, concepts, and context) or other relevant key elements used to conceptualize the review questions and/or objectives. | 4-5 |
| **METHODS** | | | |
| Protocol and registration | 5 | Indicate whether a review protocol exists; state if and where it can be accessed (e.g., a Web address); and if available, provide registration information, including the registration number. | 5 |
| Eligibility criteria | 6 | Specify characteristics of the sources of evidence used as eligibility criteria (e.g., years considered, language, and publication status), and provide a rationale. | 5-6 |
| Information sources* | 7 | Describe all information sources in the search (e.g., databases with dates of coverage and contact with authors to identify additional sources), as well as the date the most recent search was executed. | 5 |
| Search | 8 | Present the full electronic search strategy for at least 1 database, including any limits used, such that it could be repeated. | Additional file 3 |
| Selection of sources of evidence† | 9 | State the process for selecting sources of evidence (i.e., screening and eligibility) included in the scoping review. | 6 |
| Data charting process‡ | 10 | Describe the methods of charting data from the included sources of evidence (e.g., calibrated forms or forms that have been tested by the team before their use, and whether data charting was done independently or in duplicate) and any processes for obtaining and confirming data from investigators. | 6 |
| Data items | 11 | List and define all variables for which data were sought and any assumptions and simplifications made. | NA |
| Critical appraisal of individual sources of evidence§ | 12 | If done, provide a rationale for conducting a critical appraisal of included sources of evidence; describe the methods used and how this information was used in any data synthesis (if appropriate). | NA |
| Synthesis of results | 13 | Describe the methods of handling and summarizing the data that were charted. | 6 |
| **RESULTS** | | | |
| Selection of sources of evidence | 14 | Give numbers of sources of evidence screened, assessed for eligibility, and included in the review, with reasons for exclusions at each stage, ideally using a flow diagram. | 8-9 |
| Characteristics of sources of evidence | 15 | For each source of evidence, present characteristics for which data were charted and provide the citations. | 9-10, Additional file 6 |
| Critical appraisal within sources of evidence | 16 | If done, present data on critical appraisal of included sources of evidence (see item 12). | NA |
| Results of individual sources of evidence | 17 | For each included source of evidence, present the relevant data that were charted that relate to the review questions and objectives. | Additional file 6 |
| Synthesis of results | 18 | Summarize and/or present the charting results as they relate to the review questions and objectives. | 9-18 |
| **DISCUSSION** | | | |
| Summary of evidence | 19 | Summarize the main results (including an overview of concepts, themes, and types of evidence available), link to the review questions and objectives, and consider the relevance to key groups. | 19-21 |
| Limitations | 20 | Discuss the limitations of the scoping review process. | 23-24 |
| Conclusions | 21 | Provide a general interpretation of the results with respect to the review questions and objectives, as well as potential implications and/or next steps. | 24 |
| **FUNDING** | | | |
| Funding | 22 | Describe sources of funding for the included sources of evidence, as well as sources of funding for the scoping review. Describe the role of the funders of the scoping review. | 25 |

JBI = Joanna Briggs Institute; PRISMA-ScR = Preferred Reporting Items for Systematic reviews and Meta-Analyses extension for Scoping Reviews.

* Where *sources of evidence* (see second footnote) are compiled from, such as bibliographic databases, social media platforms, and Web sites.

† A more inclusive/heterogeneous term used to account for the different types of evidence or data sources (e.g., quantitative and/or qualitative research, expert opinion, and policy documents) that may be eligible in a scoping review as opposed to only studies. This is not to be confused with *information sources* (see first footnote).

‡ The frameworks by Arksey and O’Malley (6) and Levac and colleagues (7) and the JBI guidance (4, 5) refer to the process of data extraction in a scoping review as data charting*.*

§ The process of systematically examining research evidence to assess its validity, results, and relevance before using it to inform a decision. This term is used for items 12 and 19 instead of "risk of bias" (which is more applicable to systematic reviews of interventions) to include and acknowledge the various sources of evidence that may be used in a scoping review (e.g., quantitative and/or qualitative research, expert opinion, and policy document).

*From:* Tricco AC, Lillie E, Zarin W, O'Brien KK, Colquhoun H, Levac D, et al. PRISMA Extension for Scoping Reviews (PRISMAScR): Checklist and Explanation. Ann Intern Med. 2018;169:467–473. [doi: 10.7326/M18-0850](http://annals.org/aim/fullarticle/2700389/prisma-extension-scoping-reviews-prisma-scr-checklist-explanation).

# Additional file 2

Table 1. List of additional websites searched.

| ***Source*** | ***Website*** | ***Context*** |
| --- | --- | --- |
| National Institute for Health Research | https://www.nihr.ac.uk/ | UK |
| British National Health Service (NHS) | https://www.nhs.uk/ | UK |
| Royal College of Obstetricians and Gynaecologists | https://www.rcog.org.uk/ | UK |
| Tommy's | https://www.tommys.org/ | England, Scotland, Wales |
| Office for Health Improvement & Disparities | https://www.gov.uk/government/organisations/office-for-health-improvement-and-disparities | England |
| Public Health Wales | https://phw.nhs.wales/ | Wales |
| Public Health Scotland | https://www.publichealthscotland.scot/ | Scotland |
| NHS Inform | https://www.nhsinform.scot/ | Scotland |
| Health Well | https://healthwell.eani.org.uk | Northern Ireland |
| Department of Health (Northern Ireland) | https://www.health-ni.gov.uk/ | Northern Ireland |
| Sexual Health NI | https://www.sexualhealthni.info/ | Northern Ireland |
| NI DIRECT | https://www.nidirect.gov.uk/ | Northern Ireland |
| Public Health Agency | https://www.publichealth.hscni.net/ | Northern Ireland |
| Health Service Executive IE | https://www.hse.ie/eng/ | Ireland |
| Health Information and Quality Authority | https://www.hiqa.ie/areas-we-work/health-information/data-collections/all-ireland-public-health-repository | Ireland |

# Additional file 3

Table 1. Search terms used (example of Northern Ireland).

|  | Search terms |
| --- | --- |
| 1 | ~preconception AND “Northern Ireland” OR NI |
| 2 | ~preconception AND “Northern Ireland” OR NI AND policy OR service OR intervention OR guideline OR strategy OR initiative |
| 3 | “preconception health” OR “preconception care” AND Northern Ireland OR NI |
| 4 | ~pre-pregnancy AND “Northern Ireland” OR NI AND policy OR service OR intervention OR guideline OR strategy OR initiative |
| 5 | ~preconception AND Northern Ireland OR NI AND ~charity |
| 6 | ~preconception AND “Health and Social Care Trust” OR HSCT |
| 7 | “pregnancy planning” OR “planning a baby” OR “preparation for pregnancy” OR “preparation for parenthood” OR “pregnancy intention” OR “expecting mothers” OR “expectant mothers” OR “conceive” OR “future pregnancy” AND “Northern Ireland” |
| 8 | “reproductive health” OR “sexual health” AND “Northern Ireland” AND policy OR service OR intervention OR guideline OR strategy OR initiative |

# Additional file 4

Pre-conception care provision in NI – audit tool

Start of Block: Default Question Block

Q0
 **PRECONCEPTION HEALTH AUDIT TOOL

 What is this project about?**
 **Preconception health** describes the overall health of non-pregnant individuals of childbearing age (15-49 years), **before conception occurs**. If effectively optimised, it can improve maternal and infant outcomes and intergenerational health.

 **What did we do?**
 We carried out a scoping review to map the evidence regarding **preconception health and care from strategies, policies, guidelines, frameworks and recommendations** (e.g., websites, leaflets) in the UK and Ireland, published or updated after January 2011. The aim was to summarise the main themes and concepts underpinning the evidence, clarify the type of evidence available and identify gaps in knowledge.

 **What do we want to know next?**
 We want to expand on our preliminary results relevant to Northern Ireland. We are approaching a range of healthcare professionals and allied health professionals (e.g., midwives, obstetricians and gynaecologists, pharmacists, clinical leads) to see if they **have knowledge of the preconception health and care resources we identified in our scoping review and, if so, if they use them when delivering care to members of the public, regardless of gender, in the preconception phase (i.e., before pregnancy occurs).**
 To achieve this, we developed this audit tool not as a questionnaire or survey, but rather a tool to **inform research** and, thus, does not require ethical approval. Completion of this audit tool should not take more than 10-12 minutes. Please remember that there is no right or wrong answer! 

End of Block: Default Question Block

Start of Block: Block 0: role

Q1 What is your professional role?

- Midwife (1)
- Pharmacist (2)
- Obstetrician or gynaecologist (3)
- GP (4)
- Other, please specify (5) __________________________________________________

End of Block: Block 0: role

Start of Block: Block 1: NICE

Q2 Below are the main **NICE guidelines** that address preconception health and care found through our scoping review.
 Are you aware of them and do you routinely use them to inform your professional work in relation to preconception or pre-pregnancy care (i.e., **before conceiving a pregnancy**)?

|  | I am aware and routinely use in preconception care (1) | I am aware but do **not** routinely use in preconception care (2) | I am **not** aware (3) |
| --- | --- | --- | --- |
| Pre-conception - advice and management (Clinical Knowledge Summary, CKS) (1) |  |  |  |
| Infertility (CKS) (2) |  |  |  |
| Fertility problems: quality standard (3) |  |  |  |
| Fertility problems: assessment and treatment (4) |  |  |  |
| Maternal and child nutrition (5) |  |  |  |

Q3 Are there any other NICE guidelines that address preconception health and care that you are aware of and/or routinely use in your professional work? Please leave details (e.g., title) below.

________________________________________________________________

| Page Break |  |
| --- | --- |

Q4 Below are examples of **specialist NICE guidelines** that also address preconception health and care found through our scoping review. Are you aware of them and do you routinely use them to inform your professional work in relation to preconception or pre-pregnancy care (i.e., **before conceiving a pregnancy**)?

|  | I am aware and routinely use in preconception care (1) | I am aware but do **not** routinely use in preconception care (3) | I am **not** aware (2) |
| --- | --- | --- | --- |
| NICE specialist guidelines, for example: Diabetes in pregnancy: management from preconception to the postnatal period, Bipolar disorder: assessment and management, Hypertension in pregnancy, Hypothyroidism, Antenatal and postnatal mental health, Eating disorders: recognition and treatment, Endometriosis: diagnosis and management, Familial hypercholesterolaemia: identification and management, Multiple sclerosis in adults: management, Epilepsies in children, young people and adults. (1) |  |  |  |

Display This Question:

If Below are examples of specialist NICE guidelines that also address preconception health and care... = I am aware and routinely use in preconception care

Q4 Which guidelines from the list provided are you **aware of and routinely use** in preconception care?
 Examples provided:
 Diabetes in pregnancy: management from preconception to the postnatal period,
 Bipolar disorder: assessment and management,
 Hypertension in pregnancy,
 Hypothyroidism,
 Antenatal and postnatal mental health,
 Eating disorders: recognition and treatment,
 Endometriosis: diagnosis and management,
 Familial hypercholesterolaemia: identification and management,
 Multiple sclerosis in adults: management, Epilepsies in children, young people and adults.

________________________________________________________________

Display This Question:

If Below are examples of specialist NICE guidelines that also address preconception health and care... = I am aware but do not routinely use in preconception care

Q4 Which guidelines from the list provided are you **aware of but do not routinely use** in preconception care?
 Examples provided:
 Diabetes in pregnancy: management from preconception to the postnatal period,
 Bipolar disorder: assessment and management,
 Hypertension in pregnancy,
 Hypothyroidism,
 Antenatal and postnatal mental health,
 Eating disorders: recognition and treatment,
 Endometriosis: diagnosis and management,
 Familial hypercholesterolaemia: identification and management,
 Multiple sclerosis in adults: management,
 Epilepsies in children, young people and adults.

________________________________________________________________

End of Block: Block 1: NICE

Start of Block: Block 2: Royal colleges

Q5 Below are **guides from the Royal College of Obstetricians and Gynaecologists** (RCOG) that address preconception health and care found through our scoping review. Are you aware of them and do you routinely use them to inform your professional work in relation to preconception or pre-pregnancy care (i.e., **before conceiving a pregnancy**)?

|  | I am aware and routinely use in preconception care (1) | I am aware but do **not** routinely use in preconception care (3) | I am **not** aware (2) |
| --- | --- | --- | --- |
| RCOG guidelines: Care of Women with Obesity in Pregnancy Green-top Guideline No. 72, Management of Beta Thalassaemia in Pregnancy Green-top Guideline No. 66, Epilepsy in Pregnancy Green-top Guideline No. 68, Management of Women with Mental Health Issues during Pregnancy and the Postnatal Period Good Practice No.14, Pregnancy and Breast Cancer Green-top Guideline No. 12, Management of Sickle Cell Disease in Pregnancy Green-top Guideline No. 61 (1) |  |  |  |

Display This Question:

If Below are guides from the Royal College of Obstetricians and Gynaecologists (RCOG) that address p... = I am aware and routinely use in preconception care

Q5 Which guides from the list provided are you **aware of and routinely use** in preconception care? 
 Examples provided: 
 Care of Women with Obesity in Pregnancy Green-top Guideline No. 72,
 Management of Beta Thalassaemia in Pregnancy Green-top Guideline No. 66,
 Epilepsy in Pregnancy Green-top Guideline No. 68,
 Management of Women with Mental Health Issues during Pregnancy and the Postnatal Period Good Practice No.14,
 Pregnancy and Breast Cancer Green-top Guideline No. 12,
 Management of Sickle Cell Disease in Pregnancy Green-top Guideline No. 61

________________________________________________________________

Display This Question:

If Below are guides from the Royal College of Obstetricians and Gynaecologists (RCOG) that address p... = I am aware but do not routinely use in preconception care

Q5 Which guides from the list provided are you **aware of but do not routinely** use in preconception care?
 Examples provided: 
 Care of Women with Obesity in Pregnancy Green-top Guideline No. 72,
 Management of Beta Thalassaemia in Pregnancy Green-top Guideline No. 66,
 Epilepsy in Pregnancy Green-top Guideline No. 68,
 Management of Women with Mental Health Issues during Pregnancy and the Postnatal Period Good Practice No.14,
 Pregnancy and Breast Cancer Green-top Guideline No. 12,
 Management of Sickle Cell Disease in Pregnancy Green-top Guideline No. 61

________________________________________________________________

End of Block: Block 2: Royal colleges

Start of Block: Block 10

Q6 Below are **guides from other Royal colleges** and **other relevant guides** that address preconception health and care found through our scoping review.

Are you aware of them and do you routinely use them to inform your professional work in relation to preconception or pre-pregnancy care (i.e., **before conceiving a pregnancy**)?

|  | I am aware and routinely use in preconception care (1) | I am aware but do **not** routinely use in preconception care (2) | I am **not** aware (3) |
| --- | --- | --- | --- |
| Royal College of Paediatrics and Child Health (RCpreconception health) specialist guidance: Guidance Document on Valproate Use in Women and Girls of Childbearing Years (1) |  |  |  |
| Royal College of Psychiatrists (RCPSYCH) specialist guidance: Withdrawal of, and alternatives to, valproate-containing medicines in girls and women of childbearing potential who have a psychiatric illness (2) |  |  |  |
| Public Health Agency (PHA) guideline: Folic acid and vitamin D guidelines for health professionals (3) |  |  |  |
| Faculty of Sexual and Reproductive Healthcare (FSRH) specialist guide: Contraceptive Choices for Women with Cardiac Disease (4) |  |  |  |

Q7 Are there any other guides that address preconception health and care that you are aware of and/or routinely use in your professional work? Please leave details (e.g., title, source) below.

________________________________________________________________

End of Block: Block 10

Start of Block: Block 3: policies and strategies

Q8 Below are **policies and strategies** that address preconception health and care found through our scoping review.
 Are you aware of them and do you routinely use them to inform your professional work in relation to preconception or pre-pregnancy care (i.e., **before conceiving a pregnancy**)?

|  | I am aware and routinely use in preconception care (1) | I am aware but do **not** routinely use in preconception care (2) | I am **not** aware (3) |
| --- | --- | --- | --- |
| A maternity strategy for Northern Ireland 2012-2018 (1) |  |  |  |
| Review of A Strategy for Maternity Care in Northern Ireland 2012-2018 (2) |  |  |  |
| Proposal to add folic acid to flour: consultation response and consultation document (3) |  |  |  |

Q9 Are there any other policies or strategies that address preconception health and care that you are aware of and/or routinely use in your professional work? Please leave details (e.g., title, source) below.

________________________________________________________________

End of Block: Block 3: policies and strategies

Start of Block: Block 4: reports

Q10 Below are relevant **reports and other resources** that address preconception health and care found through our scoping review. Are you aware of them and do you routinely use them to inform our professional work in relation to preconception or pre-pregnancy care (i.e., **before conceiving a pregnancy**)?

|  | I am aware and routinely use in preconception care (1) | I am aware but do **not** routinely use in preconception care (2) | I am **not** aware (3) |
| --- | --- | --- | --- |
| Public Health Agency (PHA) Children's Health in Northern Ireland reports (1) |  |  |  |
| Director of Public Health Annual reports (2) |  |  |  |
| The National Institute for Health and Care Research (NIHR) Better Beginnings: Improving Health for Pregnancy (3) |  |  |  |
| MBRRACE UK Saving Lives, Improving Mothers’ Care (4) |  |  |  |
| RCOG reports, including Better for women and Providing quality care for women (5) |  |  |  |
| Faculty of Sexual and Reproductive Healthcare (FSRH) Statement from the Clinical Effectiveness Unit - Pre-conception Care (6) |  |  |  |
| British Medical Association (BMA) Alcohol and pregnancy: Preventing and managing fetal alcohol spectrum disorders (7) |  |  |  |
| Reports on specific health conditions, such as Antiepileptic drugs: review of safety of use during pregnancy by Medicines and Healthcare products Regulatory Agency (MHRA), and A Diabetes Strategic Framework by MAKING LIFE BETTER (8) |  |  |  |
| Reports on mental health issues, such as Perinatal mental health services: Recommendations for the provision of services for childbearing women by the Royal College of Psychiatrists (RCPSYCH) (9) |  |  |  |
| Regulation and Quality Improvement Authority (RQIA) reviews of services, such as the Review of Perinatal Mental Health Services in Northern Ireland, and Review of Specialist Sexual Health Services in Northern Ireland (10) |  |  |  |

Q11 Are there any other reports that address preconception health and care that you are aware of and/or routinely use in your professional work? Please leave details (e.g., title, source, url) below.

________________________________________________________________

End of Block: Block 4: reports

Start of Block: Block 5: e-learning

Q12 Below are **e-learning resources** **from the NHS platform E-Learning For Healthcare** (E-LFH), that address aspects of preconception health and care found through our scoping review. Are you aware of them, have you completed them and, if so, do you routinely use them to inform your professional work in relation to preconception or pre-pregnancy care (i.e., **before conceiving a pregnancy**)?

|  | I am aware and routinely use in preconception care (1) | I have completed and routinely use in preconception care (2) | I have completed but do **not** routinely use in preconception care (3) | I am **not** aware (4) |
| --- | --- | --- | --- | --- |
| Sexual and Reproductive Healthcare programme (1) |  |  |  |  |
| Healthy Pregnancy Pathway (2) |  |  |  |  |

Q13 Are there any other e-learning resources that address preconception health and care that you are aware of and/or routinely use in your professional work since completing them? Please leave details (e.g., title, source, url) below.

________________________________________________________________

End of Block: Block 5: e-learning

Start of Block: Block 6: Public facing: websites

Q14 Below are **web pages from the NHS website**that address preconception health and care found through our scoping review. Are you aware of them and do you routinely use them to inform your professional work in relation to preconception or pre-pregnancy care (i.e., **before conceiving a pregnancy**), for example to signpost members of the public?

|  | I am aware and routinely use in preconception care (1) | I am aware but do **not** routinely use in preconception care (2) | I am **not** aware (3) |
| --- | --- | --- | --- |
| Trying to get pregnant (1) |  |  |  |
| How can I improve my chances of becoming a dad? (2) |  |  |  |
| Infertility (3) |  |  |  |
| Pregnancy, breastfeeding and fertility while taking folic acid (4) |  |  |  |
| Do I need vitamin supplements? (5) |  |  |  |
| Vitamins, supplements and nutrition in pregnancy (6) |  |  |  |

| Page Break |  |
| --- | --- |

Q15 Below are other **web pages from the NHS website**that address preconception health and care in people with pre-existing conditions found through our scoping review. Are you aware of them and do you routinely use them to inform your professional work in relation to preconception or pre-pregnancy care (i.e., **before conceiving a pregnancy**), for example to signpost members of the public?

|  | I am aware and routinely use in preconception care (1) | I am aware but do **not** routinely use in preconception care (2) | I am **not** aware (3) |
| --- | --- | --- | --- |
| Epilepsy and pregnancy (1) |  |  |  |
| Diabetes and pregnancy (2) |  |  |  |
| Coronary heart disease and pregnancy (3) |  |  |  |
| Autosomal recessive polycystic kidney disease (4) |  |  |  |

| Page Break |  |
| --- | --- |

Q16 Below are other relevant **websites** that address preconception health and care found through our scoping review.
Are you aware of them and do you routinely use them to inform your professional work in relation to preconception or pre-pregnancy care (i.e., **before conceiving a pregnancy**), for example to signpost members of the public?

|  | I am aware and routinely use in preconception care (1) | I am aware but do **not** routinely use in preconception care (2) | I am **not** aware (3) |
| --- | --- | --- | --- |
| Advice on conceiving and preparing for pregnancy, by NI Direct (1) |  |  |  |
| Other NI Direct web pages, for example those relating to Alcohol, smoking and drugs in pregnancy, Folic Acid, Spina bifida, Healthy eating in pregnancy, and Infertility (2) |  |  |  |
| Preparing For Pregnancy and Conception Advice, by HealthWell NI (3) |  |  |  |
| Other HealthWell NI web pages, for example those relating to Alcohol & Smoking In Pregnancy, Epilepsy in Pregnancy, and Health During Pregnancy (4) |  |  |  |
| Specialist webpages from Diabetes UK, for example Planning for a pregnancy when you have diabetes and Preconception care for women with diabetes (5) |  |  |  |
| Lifestyle factors and pregnancy, by Sexual Health NI (6) |  |  |  |
| Food supplements, by SAFEFOOD (7) |  |  |  |
| Pregnancy, by SAFEFOOD (8) |  |  |  |
| Planning a pregnancy, by SEXWISE (9) |  |  |  |
| Preparing for pregnancy, by SEXWISE (10) |  |  |  |
| Before Pregnancy, on the Northern HSCT web page (11) |  |  |  |

Q17 Are there any other relevant websites that address preconception health and care that you are aware of and/or routinely use in your professional work? Please leave details (e.g., title, source, url) below.

________________________________________________________________

End of Block: Block 6: Public facing: websites

Start of Block: Block 7: Public facing, leaflets

Q18 Below are examples of **leaflets for the public** from Royal College of Obstetricians and Gynaecologists (RCOG)  that address preconception health and care found through our scoping review. Are you aware of them and do you routinely use them to inform your professional work in relation to preconception or pre-pregnancy care (i.e., **before conceiving a pregnancy**), for example to signpost members of the public?

|  | I am aware and routinely use in preconception care (1) | I am aware but do **not** routinely use in preconception care (3) | I am **not** aware (2) |
| --- | --- | --- | --- |
| RCOG leaflets, for example: Healthy eating and vitamin supplements in pregnancy, Alcohol and pregnancy, Beta thalassaemia and pregnancy, HIV and pregnancy, Pregnancy and breast cancer, Sickle cell disease and pregnancy, Smoking and pregnancy (1) |  |  |  |

Display This Question:

If Below are examples of leaflets for the public from Royal College of Obstetricians and Gynaecologi... = I am aware and routinely use in preconception care

Q18 Please state which ones from the list provided you are **aware of and routinely use** in preconception care.
 Examples provided:
 Healthy eating and vitamin supplements in pregnancy,
 Alcohol and pregnancy,
 Beta thalassaemia and pregnancy,
 HIV and pregnancy,
 Pregnancy and breast cancer,
 Sickle cell disease and pregnancy,
 Smoking and pregnancy

________________________________________________________________

Display This Question:

If Below are examples of leaflets for the public from Royal College of Obstetricians and Gynaecologi... = I am aware but do not routinely use in preconception care

Q18 Please state which ones from the list provided you are **aware of but do not routinely use** in preconception care.
 Examples provided:
 Healthy eating and vitamin supplements in pregnancy,
 Alcohol and pregnancy,
 Beta thalassaemia and pregnancy,
 HIV and pregnancy,
 Pregnancy and breast cancer,
 Sickle cell disease and pregnancy,
 Smoking and pregnancy

________________________________________________________________

End of Block: Block 7: Public facing, leaflets

Start of Block: Block 9: Other leaflets

Q19 Below are other**leaflets, toolkits and other resources for the public** that address preconception health and care found through our scoping review. Are you aware of them and do you routinely use them to inform your professional work in relation to preconception or pre-pregnancy care (i.e., **before conceiving a pregnancy**), for example to signpost members of the public?

|  | I am aware and routinely use in preconception care (1) | I am aware but do **not** routinely use in preconception care (2) | I am **not** aware (3) |
| --- | --- | --- | --- |
| Royal College of Psychiatrists (RCPSYCH) leaflets, including Planning a pregnancy, Valproate in women and girls who could get pregnant (1) |  |  |  |
| Bumps leaflets, including ones on Folic acid, Cigarettes, Sodium valproate, Zika virus in pregnancy, Paternal Exposures, Alcohol (2) |  |  |  |
| Other specialist resources, including Lupus: A Guide to pregnancy by Lupus UK, Valproate by the Medicines and Healthcare products Regulatory Agency (MHRA), Your guide to pregnancy and fertility in thyroid disorders by the British Thyroid Foundation (BTF) (3) |  |  |  |
| Pregnancy Toolkit, by Juvenile Diabetes Research Foundation (JDRF) (4) |  |  |  |
| Your sexual health: Where to go for help and advice, by the Family Planning Association (FPA) (5) |  |  |  |

Q20 Are there any other leaflets or resources for the public that address preconception health and care that you are aware of and/or routinely use in your professional work? Please leave details (e.g., title, source) below.

________________________________________________________________

End of Block: Block 9: Other leaflets

Start of Block: Block 7

Q21 Please leave any further comments you would like to add regarding resources and services that address preconception health and care in Northern Ireland.

________________________________________________________________

| Page Break |  |
| --- | --- |

Q22 If you would like to receive updates regarding the scoping review and/or if you are interested in taking part in subsequent research studies linked to preconception health and care, please leave your contact details below. Thank you in advance for your help!

________________________________________________________________

End of Block: Block 7

# Additional file 5

GRIPP2 Short form

| Section and topic | Item | Reported on page No |
| --- | --- | --- |
| 1: Aim | Report the aim of PPI in the study | 7 |
| 2: Methods | Provide a clear description of the methods used for PPI in the study | 7 |
| 3: Study results | Outcomes—Report the results of PPI in the study, including both positive and negative outcomes | 19 |
| 4: Discussion and conclusions | Outcomes—Comment on the extent to which PPI influenced the study overall. Describe positive and negative effects | 23 |
| 5: Reflections/critical perspective | Comment critically on the study, reflecting on the things that went well and those that did not, so others can learn from this experience | 23 |

PPI=patient and public involvement

# Additional file 6

Table 1. Included resources.

| ***#*** | ***Title & link*** | ***Source*** | ***Date accessed*** | ***Date published/last updated/modified (where applicable)*** | ***Context/***  ***location*** |
| --- | --- | --- | --- | --- | --- |
| 1 | 10 steps to a healthy weight > http://www.wales.nhs.uk/sitesplus/documents/888/10%20steps%20to%20a%20healthy%20weight%20E+W.pdf | AGS | 04-May-22 | nd | Wales |
| 2 | A Diabetes Strategic Framework > https://www.health-ni.gov.uk/sites/default/files/publications/health/diabetes-framework-november-2016.pdf | Additional searches | 13-Jun-22 | 2016 | NI |
| 3 | A Fitter Future for All - Framework for preventing and addressing overweight and obesity in Northern Ireland 2012-2022 > https://www.health-ni.gov.uk/sites/default/files/publications/dhssps/obesity-fitter-future-framework-ni-2012-22.pdf | AGS | 27-Apr-22 | 2012 | NI |
| 4 | A guide to fertility > https://www.britishfertilitysociety.org.uk/wp-content/uploads/2019/10/A-Guide-to-Fertility-29-10-19.pdf | Additional searches | 13-Jun-22 | 2019 | UK |
| 5 | A guide to planning for your pregnancy with PKU > https://metabolic.ie/wp-content/uploads/2015/05/A-Guide-to-Planning-a-Pregnancy-with-PKU.pdf | AGS | 07-May-22 | 2015 | Ireland |
| 6 | A guide to smoking cessation in Scotland 2010 Planning and providing specialist smoking cessation services Updated 2017 > http://www.healthscotland.scot/media/1096/a-guide-to-smoking-cessation-in-scotland-2017.pdf | Additional searches | 12-May-22 | 2017 | Scotland |
| 7 | A Healthier Future – Scotland’s Diet & Healthy Weight Delivery Plan > https://www.gov.scot/binaries/content/documents/govscot/publications/strategy-plan/2018/07/healthier-future-scotlands-diet-healthy-weight-delivery-plan/documents/00537708-pdf/00537708-pdf/govscot%3Adocument/00537708.pdf | AGS | 04-May-22 | 2018 | Scotland |
| 8 | A Healthy Weight for Ireland > https://assets.gov.ie/10073/ccbd6325268b48da80b8a9e5421a9eae.pdf | Additional Searches | 10-May-22 | 2016 | Ireland |
| 9 | A maternity strategy for Northern Ireland 2012-2018 > https://www.health-ni.gov.uk/publications/strategy-maternity-care-northern-ireland-2012-2018 | AGS | 23-Apr-22 | 2012 | NI |
| 10 | A Practical Guide to Integrated Type 2 Diabetes Care > https://www.hse.ie/eng/services/list/2/primarycare/east-coast-diabetes-service/management-of-type-2-diabetes/diabetes-and-pregnancy/icgp-guide-to-integrated-type-2.pdf | Additional searches | 12-May-22 | 2016 | Ireland |
| 11 | A Refreshed Framework for Maternity Care in Scotland the Maternity Services Action Group > https://www.gov.scot/binaries/content/documents/govscot/publications/strategy-plan/2011/02/refreshed-framework-maternity-care-scotland-maternity-services-action-group/documents/0113609-pdf/0113609-pdf/govscot%3Adocument/0113609.pdf | AGS | 04-May-22 | 2011 | Scotland |
| 12 | A report by the all-party parliamentary group on a fit and healthy childhood maternal obesity > https://fhcappg.org.uk/wp-content/uploads/2018/04/moreportfinal_june2017.pdf | Additional searches | 27-Apr-22 | 2017 | UK |
| 13 | ACE inhibitors and angiotensin II receptor antagonists: not for use in pregnancy > https://www.gov.uk/drug-safety-update/ace-inhibitors-and-angiotensin-ii-receptor-antagonists-not-for-use-in-pregnancy | Additional searches | 12-May-22 | 2014 | England |
| 14 | Advice on conceiving and preparing for pregnancy > https://www.nidirect.gov.uk/articles/advice-conceiving-and-preparing-pregnancy | AGS | 11-May-22 | nd | NI |
| 15 | Age and trying for a baby > https://www2.hse.ie/wellbeing/pregnancy-and-birth/trying-for-a-baby/age/ | Additional searches | 13-May-22 | 2019 | Ireland |
| 16 | Alcohol & Smoking In Pregnancy > https://healthwell.eani.org.uk/healthtopic/womens-health/alcohol-smoking-pregnancy | Additional searches | 11-May-22 | nd | NI |
| 17 | Alcohol > https://www.medicinesinpregnancy.org/Medicine--pregnancy/Alcohol/ | Additional searches | 06-May-22 | 2019 | UK |
| 18 | Alcohol and pregnancy > https://www.rcog.org.uk/for-the-public/browse-all-patient-information-leaflets/alcohol-and-pregnancy/ | Additional searches | 11-May-22 | 2024 | UK |
| 19 | Alcohol and pregnancy Preventing and managing fetal alcohol spectrum disorders > https://www.bma.org.uk/media/2082/fetal-alcohol-spectrum-disorders-report-feb2016.pdf | AGS | 23-Apr-22 | 2016 | UK |
| 20 | Alcohol, smoking and drugs in pregnancy > https://www.nidirect.gov.uk/articles/alcohol-smoking-and-drugs-pregnancy | Additional searches | 11-May-22 | nd | NI |
| 21 | Anaemia: migrant health guide > https://www.gov.uk/guidance/anaemia-migrant-health-guide | Additional searches | 12-May-22 | 2021 | England |
| 22 | Annual Report of the Chief Medical Officer, The Health of the 51%: Women > https://aso.org.uk/sites/default/files/page/2021-03/CMO-Report-2014.pdf | AGS | 25/04/2022 | 2014 | UK |
| 23 | Antenatal and postnatal mental health Quality standard [QS115] / Quality statement 2: Pre‑conception information > https://www.nice.org.uk/guidance/qs115/chapter/Quality-statement-2-Preconception-information | NICE | 30-Apr-22 | 2022 | UK |
| 24 | Antenatal and postnatal mental health: clinical management and service guidance > https://www.nice.org.uk/guidance/cg192/resources/antenatal-and-postnatal-mental-health-clinical-management-and-service-guidance-pdf-35109869806789 | NICE | 02-May-22 | 2020 | UK |
| 25 | Antiepileptic drugs: review of safety of use during pregnancy > https://www.gov.uk/government/publications/public-assesment-report-of-antiepileptic-drugs-review-of-safety-of-use-during-pregnancy/antiepileptic-drugs-review-of-safety-of-use-during-pregnancy | Additional searches | 14-May-22 | 2021 | UK |
| 26 | Are you getting enough? > https://www.sbhscotland.org.uk/files/Folic-Acid-2013-email-version.pdf | AGS | 04-May-22 | 2013 | Scotland |
| 27 | Are you thinking of having a baby? > https://www.england.nhs.uk/improvement-hub/wp-content/uploads/sites/44/2017/11/SAFER-Leaflet.pdf | AGS | 11-May-22 | 2011 | England |
| 28 | Before Pregnancy > http://www.northerntrust.hscni.net/services/maternity-services/before-pregnancy-2/ | AGS | 27-Apr-22 | nd | NI |
| 29 | Being overweight in pregnancy and after birth > https://www.rcog.org.uk/for-the-public/browse-all-patient-information-leaflets/being-overweight-in-pregnancy-and-after-birth/ | Additional searches | 11-May-22 | 2022 | UK |
| 30 | Best Start Strategy >https://www.nottinghamshire.gov.uk/media/2904217/nottinghamshire-best-start-strategy-2021-2025.pdf | Additional searches | 12-May-22 | 2021 | England |
| 31 | Beta thalassaemia and pregnancy patient information leaflet > https://www.rcog.org.uk/for-the-public/browse-all-patient-information-leaflets/beta-thalassaemia-and-pregnancy-patient-information-leaflet/ | Additional searches | 11-May-22 | 2022 | UK |
| 32 | Better Beginnings Improving Health for Pregnancy > https://www.uhs.nhs.uk/Media/SUHTInternet/Services/Maternity/Better-beginnings-Improving-health-for-pregnancy.pdf | AGS | 23-Apr-22 | 2017 | UK |
| 33 | Better Births Four Years On: A review of progress > https://www.england.nhs.uk/wp-content/uploads/2020/03/better-births-four-years-on-progress-report.pdf | AGS | 09-May-22 | 2020 | England |
| 34 | Better births - Improving outcomes of maternity services in England > https://www.england.nhs.uk/wp-content/uploads/2016/02/national-maternity-review-report.pdf | AGS | 10-May-22 | 2017 | England |
| 35 | Better for women > https://www.rcog.org.uk/globalassets/documents/news/campaigns-and-opinions/better-for-women/better-for-women-full-report.pdf; https://www.rcog.org.uk/media/h3smwohw/better-for-women-full-report.pdf | AGS | 23-Apr-22 | 2019 | UK |
| 36 | Bipolar disorder, pregnancy and childbirth > https://www.bipolaruk.org/Handlers/Download.ashx?IDMF=fd8688dc-309e-4afd-8386-1bbef3f814da | Additional Searches | 04-May-22 | 2014 | Wales |
| 37 | Bipolar disorder: assessment and management > https://www.nice.org.uk/guidance/cg185/resources/bipolar-disorder-assessment-and-management-pdf-35109814379461 | NICE | 02-May-22 | 2020 | UK |
| 38 | Brugada syndrome > https://www.nhs.uk/conditions/brugada-syndrome/ | Additional searches | 12-May-22 | 2020 | UK |
| 39 | Cardiovascular disease: risk assessment and reduction, including lipid modification > https://www.nice.org.uk/guidance/cg181/resources/cardiovascular-disease-risk-assessment-and-reduction-including-lipid-modification-pdf-35109807660997 | NICE | 02-May-22 | 2016 | UK |
| 40 | Care of Women with Obesity in Pregnancy > https://obgyn.onlinelibrary.wiley.com/doi/epdf/10.1111/1471-0528.15386 | Additional searches | 30-Apr-22 | 2018 | UK |
| 41 | Causes of fertility problems > https://www2.hse.ie/conditions/fertility-problems-treatments/causes/ | AGS | 06-May-22 | 2019 | Ireland |
| 42 | Children's Health in Northern Ireland > https://www.publichealth.hscni.net/sites/default/files/2019-12/RUAG%20Childrens%20Health%20in%20NI%20-%202018-19%20-%20Dec%202019.pdf | AGS | 23-Apr-22 | 2019 | NI |
| 43 | Cigarettes > https://www.medicinesinpregnancy.org/Templates/Pages/BumpsPIL.aspx?id=98318&epslanguage=en&print=y | Additional searches | 06-May-22 | 2011 | UK |
| 44 | Clinical practice guideline Nutrition During Pregnancy > https://www.hse.ie/eng/about/who/acute-hospitals-division/woman-infants/clinical-guidelines/nutrition-during-pregnancy.pdf | AGS | 07-May-22 | 2019 | Ireland |
| 45 | Clinical practice guideline the manangement of hypertension in pregnancy > https://rcpi-live-cdn.s3.amazonaws.com/wp-content/uploads/2017/02/Hypertension-Guideline_approved_120716-1.pdf | AGS | 07-May-22 | 2019 | Ireland |
| 46 | Clinical practice guideline venous thromboprophylaxis in pregnancy > https://www.hse.ie/eng/services/publications/clinical-strategy-and-programmes/venous-thromboprophylaxis-in-pregnancy.pdf | Additional searches | 13-May-22 | 2016 | Ireland |
| 47 | COMPASS Therapeutic Notes on the Management of Chronic Conditions in Pregnancy and Breastfeeding > https://www.medicinesni.com/assets/COMPASS/chronicinpregnancy.pdf | AGS | 25/04/2022 | 2014 | NI |
| 48 | Consistent messaging to promote a healthier weight > https://khub.net/documents/135939561/262823140/A+healthier+weight+-+Preconception+and+maternity+December+2019.pdf/2ebb148d-d97d-b6f8-39f6-9597bc2204da?t=1576511749741 | AGS | 10-May-22 | 2019 | England |
| 49 | Contraceptive Choices for Women with Cardiac Disease June 2014 > file:///C:/Users/40345946/Downloads/ceuguidancecontraceptivechoiceswomencardiacdisease.pdf | Additional Searches | 10-May-22 | 2014 | UK |
| 50 | Coronary heart disease and pregnancy > https://www.nhs.uk/pregnancy/related-conditions/existing-health-conditions/coronary-heart-disease/ | Additional searches | 13-May-22 | 2021 | UK |
| 51 | Coronavirus (COVID-19) Infection in Pregnancy > https://www.rcog.org.uk/media/xsubnsma/2022-03-07-coronavirus-covid-19-infection-in-pregnancy-v15.pdf | Additional searches | 11-May-22 | 2022 | UK |
| 52 | Creating a better future together: National Maternity Strategy 2016-2026 > https://assets.gov.ie/18835/ac61fd2b66164349a1547110d4b0003f.pdf | AGS | 06-May-22 | 2016 | Ireland |
| 53 | Crohn’s disease: management > https://www.nice.org.uk/guidance/ng129 | NICE | 02-May-22 | 2019 | UK |
| 54 | Delivering preconception care to women of childbearing age with serious mental illness > https://www.tommys.org/sites/default/files/2022-01/Pre-conception%20care%20and%20serious%20mental%20illness%20FINAL_updated_0.pdf | AGS | 06-May-22 | 2020 | England |
| 55 | Diabetes and Pregnancy > https://www.glucomen.co.uk/wp-content/themes/glucomen/assets/pdfs/Pregnancy_Web.pdf | AGS | 23-Apr-22 | 2021 | UK |
| 56 | Diabetes and pregnancy > https://www.nhs.uk/pregnancy/related-conditions/existing-health-conditions/diabetes/ | Additional searches | 12-May-22 | 2021 | UK |
| 57 | Diabetes and pregnancy > https://www2.hse.ie/conditions/diabetes-and-pregnancy/ | Additional searches | 13-May-22 | 2021 | Ireland |
| 58 | Diabetes and pregnancy planning NHS Royal Berkshire > https://intranet.royalberkshire.nhs.uk/patient-information-leaflets/Maternity/Maternity---diabetes-and-pregnancy-planning.htm | Additional searches | 12-May-22 | 2021 | England |
| 59 | Diabetes in pregnancy > https://111.wales.nhs.uk/doityourself/pregnancy/existinghealthproblemsdiabetes/ | Additional searches | 12-May-22 | 2021 | Wales |
| 60 | Diabetes in pregnancy Quality standard > https://www.nice.org.uk/guidance/qs109/resources/diabetes-in-pregnancy-pdf-75545246042053 | NICE | 02-May-22 | 2016 | UK |
| 61 | Diabetes in pregnancy: management from preconception to the postnatal period > https://www.nice.org.uk/guidance/ng3 | NICE | 30-Apr-22 | 2020 | UK |
| 62 | Diabetes pre-pregnancy clinics > https://online.hscni.net/diabetes-pre-pregnancy-clinics/ | AGS | 11-May-22 | 2021 | NI |
| 63 | Diabetes, pregnancy and breast-feeding > https://bnf.nice.org.uk/treatment-summary/diabetes-pregnancy-and-breast-feeding.html | Additional searches | 10-May-22 | nd | UK |
| 64 | Diagnosis -Autosomal recessive polycystic kidney disease > https://www.nhs.uk/conditions/autosomal-recessive-polycystic-kidney-disease-arpkd/diagnosis/ | Additional searches | 14-May-22 | 2022 | UK |
| 65 | Director of Public Health Annual Report 2017 > https://www.publichealth.hscni.net/sites/default/files/2018-11/DPH%20report%202017%20web%20version.pdf | AGS | 23-Apr-22 | 2017 | NI |
| 66 | Do I need vitamin supplements? > https://www.nhs.uk/common-health-questions/food-and-diet/do-i-need-vitamin-supplements/ | Additional searches | 13-May-22 | 2019 | UK |
| 67 | Do you want to have KIDS in the future? > file:///C:/Users/40345946/Downloads/Fertility-Education-Poster.pdf | Additional searches | 13-Jun-22 | nd | UK |
| 68 | Eating disorders: recognition and treatment NICE guideline > https://www.nice.org.uk/guidance/ng69/resources/eating-disorders-recognition-and-treatment-pdf-1837582159813 | NICE | 30-Apr-22 | 2020 | UK |
| 69 | Endocrine Late Effects > https://cdn.macmillan.org.uk/dfsmedia/1a6f23537f7f4519bb0cf14c45b2a629/1530-source/endocrine-late-effects-tcm9-340519 | AGS | 25/04/2022 | 2020 | UK |
| 70 | Endometriosis: diagnosis and management NICE guideline [NG73] > https://www.nice.org.uk/guidance/ng73 | NICE | 02-May-22 | 2017 | UK |
| 71 | Epilepsies in children, young people and adults NICE guideline [NG217] > https://www.nice.org.uk/guidance/ng217 | AGS | 30-Apr-22 | 2022 | UK |
| 72 | Epilepsy & Pregnancy > https://healthwell.eani.org.uk/healthtopic/epilepsy/epilepsy-pregnancy | Additional searches | 11-May-22 | nd | NI |
| 73 | Epilepsy > https://www.nhsinform.scot/illnesses-and-conditions/brain-nerves-and-spinal-cord/epilepsy/#living-with-epilepsy | Additional searches | 13-May-22 | 2021 | Scotland |
| 74 | Epilepsy and planning a pregnancy > https://www.tommys.org/pregnancy-information/planning-a-pregnancy/are-you-ready-to-conceive/epilepsy-and-planning-pregnancy | Additional Searches | 09-May-22 | 2021 | UK |
| 75 | Epilepsy and pregnancy > https://www.nhs.uk/pregnancy/related-conditions/existing-health-conditions/epilepsy/ | Additional searches | 11-May-22 | 2021 | UK |
| 76 | Epilepsy in Pregnancy Green-top Guideline No. 68 > https://www.rcog.org.uk/globalassets/documents/guidelines/green-top-guidelines/gtg68_epilepsy.pdf | Additional searches | 30-Apr-22 | 2016 | UK |
| 77 | Epilepsy medicines and pregnancy > https://www.gov.uk/government/publications/epilepsy-medicines-and-pregnancy | Additional Searches | 10-May-22 | 2021 | England |
| 78 | Every Baby Matters > https://www.bradford.gov.uk/media/1903/2-nutrition-guidelines-preconception.pdf | Additional Searches | 10-May-22 | 2015 | England |
| 79 | Evidence-based nutrition guidelines for the prevention and management of diabetes > https://www.diabetes.org.uk/professionals/position-statements-reports/food-nutrition-lifestyle/evidence-based-nutrition-guidelines-for-the-prevention-and-management-of-diabetes | Audit | 30-Jun-23 | 2018 | UK |
| 80 | Familial hypercholesterolaemia: identification and management > https://www.nice.org.uk/guidance/cg71 | NICE | 02-May-22 | 2019 | UK |
| 81 | Family planning and fertility > https://www.tommys.org/pregnancy-information/planning-a-pregnancy/planning-a-pregnancy-and-mental-illness/family-planning-and-fertility | Additional Searches | 09-May-22 | 2020 | UK |
| 82 | Fertility problems > https://www.nice.org.uk/guidance/qs73/resources/fertility-problems-2098846428613 | AGS | 30-Apr-22 | 2014 | UK |
| 83 | Fertility problems: assessment and treatment > https://www.nice.org.uk/guidance/cg156 | NICE | 13-May-22 | 2013 | UK |
| 84 | Fertility treatment > https://www2.hse.ie/conditions/fertility-problems-treatments/fertility-treatment/ | AGS | 06-May-22 | 2019 | Ireland |
| 85 | Fertility treatment options > https://www.tommys.org/pregnancy-information/planning-a-pregnancy/fertility-and-causes-of-infertility/fertility-treatment-options | Additional Searches | 09-May-22 | 2021 | UK |
| 86 | First 1000 days of life Thirteenth Report of Session 2017–19 > file:///C:/Users/40345946/Downloads/First%201000%20days%20of%20life%20-%20House%20of%20Commons%20Report%20Feb19.pdf | AGS | 25/04/2022 | 2019 | UK |
| 87 | First 5 A Whole-of-Government Strategy for Babies, Young Children and their Families 2019-2028 > https://assets.gov.ie/31184/62acc54f4bdf4405b74e53a4afb8e71b.pdf | Additional Searches | 11-May-22 | 2019 | Ireland |
| 88 | First 5 Annual Implementation Report 2019 > https://first5.gov.ie/files/DCEDIY_AnnImRep2019_EN.pdf | Additional Searches | 11-May-22 | 2020 | Ireland |
| 89 | Fit for and during pregnancy A key role for local government > https://www.local.gov.uk/sites/default/files/documents/15.52%20Fit%20for%20and%20during%20pregnancy_03.pdf | Additional Searches | 10-May-22 | 2018 | England |
| 90 | Folic Acid > https://www.hse.ie/eng/about/who/healthwellbeing/our-priority-programmes/heal/folic-acid/ | Additional searches | 13-May-22 | nd | Ireland |
| 91 | Folic acid > https://www.medicinesinpregnancy.org/Templates/Pages/BumpsPIL.aspx?id=97949&epslanguage=en&print=y | Additional searches | 06-May-22 | 2017 | UK |
| 92 | Folic Acid > https://www.nidirect.gov.uk/articles/folic-acid | Additional searches | 11-May-22 | nd | NI |
| 93 | Folic acid > https://www2.hse.ie/conditions/folic-acid/ | Additional searches | 13-May-22 | 2020 | Ireland |
| 94 | Folic acid and Vitamin D Guidelines for Health professionals > https://www.publichealth.hscni.net/sites/default/files/FOLIC%20ACID%20and%20VITAMIN%20D%20Guidelines%202017.pdf | Additional searches | 12-May-22 | 2017 | NI |
| 95 | Folic acid when planning a pregnancy > https://www2.hse.ie/wellbeing/pregnancy-and-birth/trying-for-a-baby/food-supplements/folic-acid/ | Additional Searches | 06-May-22 | 2021 | Ireland |
| 96 | Folic acid: Before and during pregnancy > http://www.healthscotland.com/uploads/documents/5101-Folic%20acid%20-%20Before%20and%20during%20pregnancy-Feb2019-English.pdf | AGS | 06-May-22 | 2019 | Scotland |
| 97 | Food supplements > https://www.safefood.net/Healthy-Eating/What-is-a-healthy-diet-(2)/Food-supplements | Additional searches | 11-May-22 | nd | Ireland |
| 98 | Foods to eat when planning a pregnancy > https://www2.hse.ie/wellbeing/pregnancy-and-birth/trying-for-a-baby/food-supplements/foods-to-eat/ | Additional Searches | 06-May-22 | 2021 | Ireland |
| 99 | Gluten free when pregnant > https://www.coeliac.org.uk/information-and-support/living-gluten-free/the-gluten-free-diet/gluten-free-when-pregnant/?&&type=rfst&set=true#cookie-widget | Additional searches | 13-May-22 | nd | UK |
| 100 | Guidance Document on Valproate Use in Women and Girls of Childbearing Years > https://www.rcpch.ac.uk/sites/default/files/2021-01/Pan_College_Guidance_Document_on_Valproate_Use%20V2.1.pdf | Additional searches | 11-May-22 | 2020 | UK |
| 101 | Haemoglobin disorders: migrant health guide > https://www.gov.uk/guidance/haemoglobin-disorders-migrant-health-guide | Additional searches | 14-May-22 | 2014 | England |
| 102 | Health During Pregnancy > https://healthwell.eani.org.uk/node/2722 | Additional searches | 11-May-22 | nd | NI |
| 103 | Health during pregnancy > https://www.nidirect.gov.uk/articles/health-during-pregnancy#toc-0 | Additional searches | 11-May-22 | nd | NI |
| 104 | Health matters: giving every child the best start in life > https://www.gov.uk/government/publications/health-matters-giving-every-child-the-best-start-in-life/health-matters-giving-every-child-the-best-start-in-life | Additional searches | 06-May-22 | 2016 | England |
| 105 | Health matters: Prevention - a life course approach> https://www.gov.uk/government/publications/health-matters-life-course-approach-to-prevention/health-matters-prevention-a-life-course-approach | Additional Searches | 10-May-22 | 2019 | England |
| 106 | Health Matters: Reproductive health and pregnancy planning > https://ukhsa.blog.gov.uk/2018/06/26/health-matters-reproductive-health-and-pregnancy-planning/ | AGS | 09-May-22 | 2018 | England |
| 107 | Health of women before and during pregnancy: health behaviours, risk factors and inequalities > https://assets.publishing.service.gov.uk/government/uploads/system/uploads/attachment_data/file/844210/Health_of_women_before_and_during_pregnancy_2019.pdf | AGS | 10-May-22 | 2019 | England |
| 108 | Health problems -Type 2 diabetes > https://www.nhs.uk/conditions/type-2-diabetes/health-problems/ | Additional searches | 13-May-22 | 2020 | UK |
| 109 | Healthier Eating before Pregnancy > https://ihv.org.uk/wp-content/uploads/2021/04/Preconception-Nutrition-Infographic-2021-FINAL-1.pdf | Additional Searches | 10-May-22 | 2021 | UK |
| 110 | Healthy beginnings: applying All Our Health > https://www.gov.uk/government/publications/healthy-beginnings-applying-all-our-health/healthy-beginnings-applying-all-our-health | Additional Searches | 10-May-22 | 2022 | England |
| 111 | Healthy eating and vitamin supplements in pregnancy patient information leaflet > https://www.rcog.org.uk/for-the-public/browse-all-patient-information-leaflets/healthy-eating-and-vitamin-supplements-in-pregnancy-patient-information-leaflet/ OR https://www.rcog.org.uk/media/1kjbcouw/pi-healthy-eating-and-vitamin-supplements-in-pregnancy.pdf | Additional searches | 11-May-22 | 2022 | UK |
| 112 | Healthy eating in pregnancy > https://www.nidirect.gov.uk/articles/healthy-eating-pregnancy | Additional searches | 11-May-22 | nd | NI |
| 113 | Healthy Pregnancy Pathway > https://www.e-lfh.org.uk/heathy-pregnancy-pathway/index.html | AGS | 10-May-22 | nd | UK |
| 114 | Healthy weight when planning a pregnancy > https://www2.hse.ie/wellbeing/pregnancy-and-birth/trying-for-a-baby/healthy-weight/ | AGS | 06-May-22 | 2021 | Ireland |
| 115 | Hearty Lives - Mid and East Antrim Borough Council > https://www.midandeastantrim.gov.uk/downloads/Hearty_Lives_Report_12pt_Final.pdf | AGS | 25/04/2022 | 2016 | NI |
| 116 | HIV and pregnancy patient information leaflet > https://www.rcog.org.uk/for-the-public/browse-all-patient-information-leaflets/hiv-and-pregnancy-patient-information-leaflet/ | Additional searches | 11-May-22 | 2022 | UK |
| 117 | How can I improve my chances of becoming a dad? > https://www.nhs.uk/common-health-questions/mens-health/how-can-i-improve-my-chances-of-becoming-a-dad/ | Additional searches | 13-Jun-22 | 2020 | UK |
| 118 | How to improve male fertility > https://www.tommys.org/pregnancy-information/planning-a-pregnancy/are-you-ready-to-conceive/how-improve-male-fertility | Additional Searches | 09-May-22 | 2021 | UK |
| 119 | How to improve your chances of getting pregnant > https://www2.hse.ie/wellbeing/pregnancy-and-birth/trying-for-a-baby/improve-your-chances/ | AGS | 07-May-22 | 2021 | Ireland |
| 120 | Hyperparathyroidism (primary): diagnosis, assessment and initial management > https://www.nice.org.uk/guidance/ng132 | NICE | 02-May-22 | 2019 | UK |
| 121 | Hypertension in pregnancy - Quality standard [QS35] > https://www.nice.org.uk/guidance/qs35 | NICE | 02-May-22 | 2019 | UK |
| 122 | Hypertension in pregnancy: diagnosis and management > https://www.nice.org.uk/guidance/ng133/resources/hypertension-in-pregnancy-diagnosis-and-management-pdf-66141717671365 | NICE | 02-May-22 | 2019 | UK |
| 123 | hypothyroidism? > https://cks.nice.org.uk/topics/hypothyroidism/ | NICE | 30-Apr-22 | 2021 | UK |
| 278 | Immunisation Guidelines > https://www.hse.ie/eng/health/immunisation/hcpinfo/guidelines/ | Additional searches | 16/11/2022 | 2022 | Ireland |
| 125 | Implementing Better Births - A resource pack for Local Maternity Systems > https://www.england.nhs.uk/wp-content/uploads/2017/03/nhs-guidance-maternity-services-v1-print.pdf | AGS | 10-May-22 | 2017 | England |
| 126 | Improving Maternal and Infant Nutrition: A Framework for Action > https://www.gov.scot/binaries/content/documents/govscot/publications/advice-and-guidance/2011/01/improving-maternal-infant-nutrition-framework-action/documents/0110855-pdf/0110855-pdf/govscot%3Adocument/0110855.pdf | AGS | 04-May-22 | 2011 | Scotland |
| 127 | Infections > https://phw.nhs.wales/services-and-teams/caris/key-anomalies/infections/ | Additional searches | 12-May-22 | nd | Wales |
| 128 | Infertility > https://cks.nice.org.uk/topics/infertility/ | Additional searches | 30-Apr-22 | 2018 | UK |
| 129 | Infertility > https://www.nhs.uk/conditions/infertility/ | Additional searches | 12-May-22 | 2020 | UK |
| 130 | Infertility > https://www.nidirect.gov.uk/conditions/infertility | Additional searches | 11-May-22 | nd | NI |
| 131 | Infertility in men > https://www2.hse.ie/conditions/fertility-problems-treatments/infertility-men/ | AGS | 06-May-22 | 2019 | Ireland |
| 132 | Infertility in women > https://www2.hse.ie/conditions/fertility-problems-treatments/infertility-women/ | AGS | 06-May-22 | 2019 | Ireland |
| 133 | Information and choices for women and couples at risk of having a baby with thalassaemia major > https://www.gov.uk/government/publications/baby-at-risk-of-having-thalassaemia-description-in-brief/information-and-choices-for-women-and-couples-at-risk-of-having-a-child-with-thalassaemia-major#planning-for-pregnancy | Additional searches | 14-May-22 | 2021 | England |
| 134 | Interactive Townscapes, Contraception and preconception care > https://www.e-lfh.org.uk/townscape-reproductive-health/ | Additional searches | 13-May-22 | nd | England |
| 135 | Iron and calcium when planning a pregnancy > https://www2.hse.ie/wellbeing/pregnancy-and-birth/trying-for-a-baby/food-supplements/iron-calcium/ | AGS | 06-May-22 | 2021 | Ireland |
| 136 | Life stage: Pre-conception and pregnancy > https://assets.publishing.service.gov.uk/government/uploads/system/uploads/attachment_data/file/252655/33571_2901304_CMO_Chapter_5.pdf | Additional searches | 10-May-22 | 2012 | England |
| 137 | Lifestyle factors and pregnancy > https://www.sexualhealthni.info/lifestyle-factors-and-pregnancy | Additional searches | 11-May-22 | 2015 | NI |
| 138 | Lithium in Pregnancy and Breastfeeding > https://www.hse.ie/eng/services/publications/mentalhealth/lithium-in-pregnancy-and-breastfeeding.pdf | Additional searches | 12-May-22 | 2019 | Ireland |
| 139 | Living with -Psoriasis > https://www.nhs.uk/conditions/psoriasis/living-with/ | Additional searches | 13-May-22 | 2022 | UK |
| 140 | Living with -Sickle cell disease > https://www.nhs.uk/conditions/sickle-cell-disease/living-with/ | Additional searches | 13-May-22 | 2019 | UK |
| 141 | Living with Thalassaemia > https://www.nhs.uk/conditions/thalassaemia/living-with/ | Additional searches | 12-May-22 | 2022 | UK |
| 142 | Lupus A Guide to pregnancy > https://www.lupusuk.org.uk/wp-content/uploads/2015/09/LUPUS-A-Guide-to-Pregnancy-V1.pdf | Additional Searches | 09-May-22 | 2017 | UK |
| 143 | Lupus and planning a pregnancy > https://www.tommys.org/pregnancy-information/planning-pregnancy/health-conditions-and-planning-pregnancy/lupus-and-planning | Additional Searches | 09-May-22 | 2021 | UK |
| 144 | Making the case for preconception care - GOV.UK > https://assets.publishing.service.gov.uk/government/uploads/system/uploads/attachment_data/file/729018/Making_the_case_for_preconception_care.pdf | AGS | 06-May-22 | 2018 | England |
| 145 | Management of Beta Thalassaemia in Pregnancy (Green-top Guideline No. 66) > https://www.rcog.org.uk/guidance/browse-all-guidance/green-top-guidelines/management-of-beta-thalassaemia-in-pregnancy-green-top-guideline-no-66/ | Additional searches | 11-May-22 | 2014 | UK |
| 146 | Management of Inherited Bleeding Disorders in Pregnancy Green-top Guideline No.71 > https://obgyn.onlinelibrary.wiley.com/doi/pdfdirect/10.1111/1471-0528.14592 | Additional searches | 11-May-22 | 2017 | UK |
| 147 | Management of Obesity During Pregnancy Guideline > https://wisdom.nhs.wales/health-board-guidelines/hywel-dda-file/obesity-in-pregnancy-guideline-2-hywell-dda-guideline-2022-pdf/ | AGS | 04-May-22 | 2022 | Wales |
| 148 | Management of Women with Mental Health Issues during Pregnancy and the Postnatal Period (Good Practice No.14) > https://www.rcog.org.uk/guidance/browse-all-guidance/good-practice-papers/management-of-women-with-mental-health-issues-during-pregnancy-and-the-postnatal-period-good-practice-no14/ | Additional searches | 12-May-22 | 2011 | UK |
| 149 | Maternal and child nutrition > https://www.nice.org.uk/guidance/ph11/resources/maternal-and-child-nutrition-pdf-1996171502533 | NICE | 02-May-22 | 2014 | UK |
| 150 | Maternal and Child Nutrition Best Practice Guidance > http://www.forhighlandschildren.org/4-icspublication/index_6_3071689108.pdf | Additional Searches | 04-May-22 | 2011 | Scotland |
| 151 | Maternity > https://setrust.hscni.net/service/maternity-2/ | Audit | 30-Jun-23 | 2022 (update) | NI |
| 152 | Maternity high impact area: Improving planning and preparation for pregnancy > https://assets.publishing.service.gov.uk/government/uploads/system/uploads/attachment_data/file/942474/Maternity_high_impact_area_1_Improving_planning_and_preparation_for_pregnancy.pdf | AGS | 09-May-22 | 2020 | England |
| 153 | Maternity high impact area: Reducing the incidence of harms caused by alcohol in pregnancy > https://assets.publishing.service.gov.uk/government/uploads/system/uploads/attachment_data/file/942477/Maternity_high_impact_area_4_Reducing_the_incidence_of_harms_caused_by_alcohol_in_pregnancy.pdf | AGS | 10-May-22 | 2021 | England |
| 154 | Maternity high impact area: Reducing the inequality of outcomes for women from Black, Asian and Minority Ethnic (BAME) communities and their babies: https://assets.publishing.service.gov.uk/government/uploads/system/uploads/attachment_data/file/942480/Maternity_high_impact_area_6_Reducing_the_inequality_of_outcomes_for_women_from_Black__Asian_and_Minority_Ethnic__BAME__communities_and_their_babies.pdf | AGS | 10-May-22 | 2020 | England |
| 155 | Maternity high impact area: Supporting good parental mental health > https://assets.publishing.service.gov.uk/government/uploads/system/uploads/attachment_data/file/942475/Maternity_high_impact_area_2_Supporting_good_parental_mental_health.pdf | AGS | 10-May-22 | 2020 | England |
| 156 | Maternity high impact area: Supporting healthy weight before and between pregnancies > https://assets.publishing.service.gov.uk/government/uploads/system/uploads/attachment_data/file/942476/Maternity_high_impact_area_3_Supporting_healthy_weight_before_and_between_pregnancies_.pdf | Additional searches | 06-May-22 | 2020 | England |
| 157 | Maternity high impact area: Supporting parents to have a smokefree pregnancy > https://assets.publishing.service.gov.uk/government/uploads/system/uploads/attachment_data/file/942478/Maternity_high_impact_area_5_Supporting_parents_to_have_a_smokefree_pregnancy.pdf | AGS | 10-May-22 | 2020 | England |
| 158 | Measles, Mumps and Rubella (MMR) > https://phw.nhs.wales/topics/immunisation-and-vaccines/vaccines/mmr/ | Additional searches | 13-May-22 | nd | Wales |
| 159 | Medicines and Healthcare products Regulatory Agency "Valproate" > https://assets.publishing.service.gov.uk/government/uploads/system/uploads/attachment_data/file/950801/107995_Valproate_Patient_Booklet_v05_DS_07-01-2021.pdf | Additional searches | 30-Apr-22 | 2020 | UK |
| 160 | Missed Periods - Scotland’s opportunities for better pregnancies, healthier parents and thriving babies the first time ... and every time> https://www.nhsggc.org.uk/media/237840/missed-periods-j-sher-may-2016.pdf | AGS | 04-May-22 | 2016 | Scotland |
| 161 | Model of Integrated Care for Patients with Type 2 Diabetes A Guide for Health Care Professionals (Clinical Management Guidelines) > https://www.hse.ie/eng/about/who/cspd/ncps/diabetes/moc/model-of-integrated-care-type-2-diabetes-2018.pdf | Additional searches | 12-May-22 | 2018 | Ireland |
| 162 | Multiple sclerosis in adults: management (CG186) > https://www.nice.org.uk/guidance/ng220/resources/multiple-sclerosis-in-adults-management-pdf-66143828948677 | NICE | 30-Apr-22 | 2022 | UK |
| 163 | National Maternity and Perinatal Audit Ethnic and Socio-economic Inequalities in NHS Maternity and Perinatal Care for Women and their Babies > https://maternityaudit.org.uk/FilesUploaded/Ref%20308%20Inequalities%20Sprint%20Audit%20Report%202021_FINAL.pdf | Open Aire | 12/04/2022 | 2021 | UK |
| 164 | National Maternity and Perinatal Audit NHS Maternity Care for Women with a Body Mass Index of 30 kg/m2 or Above > https://maternityaudit.org.uk/FilesUploaded/NMPA%20BMI%20Over%2030%20Report.pdf | Open Aire | 13/04/2022 | 2021 | UK |
| 165 | National Practice Guide – Seeing through Hidden Harm to brighter futures > https://www.hse.ie/eng/about/who/primarycare/socialinclusion/addiction/national-addiction-training/national-hidden-harm-project/hiddenharmpracticeguide-2019.pdf | Additional searches | 13-May-22 | 2019 | Ireland |
| 166 | National Standards for Safer Better Maternity Services > https://www.hiqa.ie/sites/default/files/2017-02/national-standards-maternity-services.pdf | Additional searches | 10-May-22 | 2016 | Ireland |
| 167 | New taskforce to level-up maternity care and tackle disparities > https://www.gov.uk/government/news/new-taskforce-to-level-up-maternity-care-and-tackle-disparities | Additional Searches | 09-May-22 | 2022 | England |
| 168 | NSPCC Scotland Policy, Practice and Research Series Infant Mental Health: The Scottish Context > https://www.nspcc.org.uk/globalassets/documents/consultation-responses/nspcc-scotland-2012-briefing-infant-mental-health-policy-context.pdf | AGS | 06-May-22 | 2021 | Scotland |
| 169 | Nutrition in Pregnancy > https://www.healthprofessionalacademy.co.uk/mum-and-baby/learn/nutrition-in-pregnancy-cpd | Audit | 30-Jun-23 | nd | England, Wales (open to everyone) |
| 170 | Nutritional management of cystic fibrosis > https://www.cysticfibrosis.org.uk/sites/default/files/2020-12/Nutritional%20Management%20of%20cystic%20fibrosis%20Sep%2016.pdf | AGS | 27-Apr-22 | 2016 | UK |
| 171 | Nutritional Supplements in Pregnancy > https://www.healthprofessionalacademy.co.uk/mum-and-baby/learn/nutritional-supplements-in-pregnancy-mba | Audit | 30-Jun-23 | nd | England, Wales (open to everyone) |
| 172 | Our Vision for the Women’s Health Strategy for England > https://www.gov.uk/government/publications/our-vision-for-the-womens-health-strategy-for-england | Additional searches | 14-May-22 | 2022 | England |
| 173 | Pan-London Perinatal Mental Health Networks > https://www.healthylondon.org/wp-content/uploads/2019/05/Pre-conception-advice-Best-Practice-Toolkit-for-Perinatal-Mental-Health-Services.pdf | Additional searches | 10-May-22 | 2019 | England |
| 174 | Paternal Exposures > https://www.medicinesinpregnancy.org/Templates/Pages/BumpsPIL.aspx?id=106260&epslanguage=en&print=y | Additional searches | 07-May-22 | 2017 | UK |
| 175 | Perinatal Mental Health Care: Best Practice Principles for Midwives, Public Health Nurses and Practice Nurses > https://healthservice.hse.ie/filelibrary/mind-mothers-project.pdf | Additional searches | 12-May-22 | 2017 | Ireland |
| 176 | Perinatal mental health in Wales > https://senedd.wales/laid%20documents/cr-ld11234/cr-ld11234-e.pdf | AGS | 04-May-22 | 2017 | Wales |
| 177 | Perinatal mental health services: Recommendations for the provision of services for childbearing women > https://www.rcpsych.ac.uk/docs/default-source/improving-care/better-mh-policy/college-reports/college-report-cr232---perinatal-mental-heath-services.pdf?Status=Master&sfvrsn=82b10d7e_4 | AGS | 23-Apr-22 | 2018 | UK |
| 178 | Planning a pregnancy > https://www.rcpsych.ac.uk/mental-health/treatments-and-wellbeing/planning-a-pregnancy | AGS | 25/04/2022 | 2018 | UK |
| 179 | Planning a pregnancy > https://www.sexwise.org.uk/planning-pregnancy/planning-pregnancy | AGS | 27-Apr-22 | 2018 | UK |
| 180 | Planning a pregnancy > https://www.tommys.org/pregnancy-information/planning-pregnancy/planning-for-pregnancy-tool | Additional searches | 06-May-22 | nd | UK |
| 181 | Planning a pregnancy with type 1 or 2 diabetes > https://www.tommys.org/pregnancy-information/planning-a-pregnancy/are-you-ready-to-conceive/planning-pregnancy-type-1-or-2-diabetes | Additional Searches | 09-May-22 | 2021 | UK |
| 182 | Planning a Pregnancy: Information for women with mental health problems > https://www.hse.ie/eng/services/publications/mentalhealth/planning-a-pregnancy-information-for-women-with-mental-health-problems.pdf | Additional searches | 12-May-22 | 2019 | Ireland |
| 183 | planning for a pregnancy when you have diabetes > https://www.diabetes.org.uk/guide-to-diabetes/life-with-diabetes/pregnancy | AGS | 25/04/2022 | 2022 | UK |
| 184 | Planning for pregnancy > https://www.epilepsy.ie/content/planning-pregnancy | AGS | 07-May-22 | nd | Ireland |
| 279 | Planning pregnancy: a guide for women at high risk of Postpartum Psychosis > https://www.app-network.org/wp-content/uploads/2013/12/Planning-Pregnancy-Guide-for-Women-at-High-Risk-of-PP.pdf | Additional searches | 17/11/2022 | 2014 | Ireland |
| 186 | Planning Your Pregnancy > https://www.diabetes.ie/living-with-diabetes/living-type-1/pregnancy/planning-pregnancy/ | AGS | 06-May-22 | 2019 | Ireland |
| 187 | Planning your pregnancy > https://www.nhs.uk/pregnancy/trying-for-a-baby/planning-your-pregnancy/ | Additional searches | 06-May-22 | 2020 | England |
| 188 | Postpartum Psychosis > https://www.hse.ie/eng/services/publications/mentalhealth/postpartum-psychosis.pdf | Additional searches | 12-May-22 | 2019 | Ireland |
| 189 | Practice Guide for Management of Women with Epilepsy > https://www.hse.ie/eng/services/publications/clinical-strategy-and-programmes/practice-guide-for-mgt-of-women-with-epilepsy.pdf | Additional searches | 12-May-22 | 2018 | Ireland |
| 190 | Pre-conception - advice and management > https://cks.nice.org.uk/topics/pre-conception-advice-management/ | Additional searches | 30-Apr-22 | 2021 | UK |
| 191 | Preconception and Pregnancy > https://www.bathnes.gov.uk/sites/default/files/banes_maternal_and_child_nutrition_guidelines_digital_june_2013_section_2.pdf | Additional Searches | 10-May-22 | 2013 | UK |
| 192 | preconception care for women with diabetes (may 2015) > https://www.diabetes.org.uk/professionals/position-statements-reports/specialist-care-for-children-and-adults-and-complications/preconception-care-for-women-with-diabetes | AGS | 11-May-22 | 2015 | UK |
| 193 | Preconception Health Toolkit > https://sexualhealthdg.co.uk/downloads/Preconception%20Health%20Toolkit.pdf | AGS | 04-May-22 | 2016 | Scotland |
| 194 | Preconception interventions and resources for women with serious mental illness A Rapid Evidence Review > https://assets.publishing.service.gov.uk/government/uploads/system/uploads/attachment_data/file/1034620/phe-evidence-review-8-march.pdf | Additional searches | 10-May-22 | 2021 | England |
| 195 | PRE-CONCEPTION, Oxford University Hospitals NHS Foundation Trust > https://www.ouh.nhs.uk/maternity/pre-conception/ | Additional searches | 12-May-22 | 2022 (copyright in 2022) | England |
| 196 | Pregabalin > https://www.nhs.uk/medicines/pregabalin/ | Additional searches | 13-May-22 | 2021 | UK |
| 197 | Pregnancy > https://www.safefood.net/Healthy-Eating/Family-health/Pregnancy | Additional searches | 11-May-22 | nd | Ireland |
| 198 | Pregnancy and alcohol > https://www.nidirect.gov.uk/articles/pregnancy-and-alcohol#toc-0 | Additional searches | 11-May-22 | nd | NI |
| 199 | Pregnancy and Breast Cancer (Green-top Guideline No. 12) > https://www.rcog.org.uk/guidance/browse-all-guidance/green-top-guidelines/pregnancy-and-breast-cancer-green-top-guideline-no-12/ | Additional searches | 11-May-22 | 2011 | UK |
| 200 | Pregnancy and breast cancer patient information leaflet > https://www.rcog.org.uk/for-the-public/browse-all-patient-information-leaflets/pregnancy-and-breast-cancer-patient-information-leaflet/ | Additional searches | 11-May-22 | 2023 | UK |
| 201 | Pregnancy and giving birth > https://www.nhs.uk/conditions/type-1-diabetes/living-with-type-1-diabetes/pregnancy-and-giving-birth/ | Additional searches | 13-May-22 | 2021 | UK |
| 202 | Pregnancy and Parenthood in Young People Strategy > https://www.gov.scot/publications/pregnancy-parenthood-young-people-strategy/pages/2/ | AGS | 04-May-22 | 2016 | Scotland |
| 203 | Pregnancy Guide > https://111.wales.nhs.uk/livewell/pregnancy/SecretstosuccessPlanning/ | AGS | 04-May-22 | 2022 | Wales |
| 204 | Pregnancy In Women With Bleeding Disorders > https://haemophilia.ie/living-with-haemophilia/women-with-bleeding-disorders/pregnancy-in-women-with-bleeding-disorders/ | AGS | 06-May-22 | nd | Ireland |
| 205 | Pregnancy preconception and planning > https://www.healthprofessionalacademy.co.uk/mum-and-baby/learn/pregnancy-planning-cpd | Audit | 30-Jun-23 | nd | England, Wales (open to everyone) |
| 206 | Pregnancy Toolkit JDRF > https://nhsforthvalley.com/wp-content/uploads/2021/03/JDRF-PregnancyToolkit_21.pdf | AGS | 25/04/2022 | 2021 | UK |
| 207 | Pregnancy, breastfeeding and fertility while taking folic acid > https://www.nhs.uk/medicines/folic-acid/pregnancy-breastfeeding-and-fertility-while-taking-folic-acid/ | Additional searches | 12-May-22 | 2022 | UK |
| 208 | Prepared for Pregnancy? Preconception health, education and care in Scotland > https://www.stor.scot.nhs.uk/bitstream/handle/11289/578820/prepared-for-pregnancy-j-sher-may-2016.pdf?sequence=1&isAllowed=y | AGS | 04-May-22 | 2016 | Scotland |
| 209 | PREPARING FOR HEALTHIER PREGNANCIES, BETTER LIVES > https://vhscotland.org.uk/wp-content/uploads/2021/09/QNIS.pdf | AGS | 04-May-22 | 2021 | Scotland |
| 210 | Preparing For Pregnancy and Conception Advice > https://healthwell.eani.org.uk/node/2719 | AGS | 11-May-22 | nd | NI |
| 211 | Preparing for pregnancy> https://www.sexwise.org.uk/planning-pregnancy/preparing-pregnancy | AGS | 27-Apr-22 | 2018 | UK |
| 212 | Pre-Pregnancy Health > https://www.lanarkshiresexualhealth.org/pre-pregnancy-health/ | AGS | 13-May-22 | nd | Scotland |
| 213 | Prevention of Rubella > https://www.hse.ie/eng/health/immunisation/hcpinfo/othervaccines/rubella/engrubella.pdf | Additional searches | 13-May-22 | 2021 | Ireland |
| 214 | Primary prevention of congenital anomalies > https://eu-rd-platform.jrc.ec.europa.eu/sites/default/files/EUROCAT-EUROPLAN-Primary-Preventions-Reccomendations.pdf | AGS | 12-May-22 | multiple dates (2011-2013, 2012-2015) | Ireland (EU) |
| 215 | Promoting Scotland’s sexual and reproductive health: A joint manifesto > https://www.fsrh.org/documents/joint-manifesto-scotland-elections-sexual-reproductive-health/ | AGS | 06-May-22 | 2021 | Scotland |
| 216 | Proposal to add folic acid to flour: consultation document > https:// www.gov.uk/government/consultations/adding-folic-acid-to-flour/proposal-to-add-folic-acid-to-flour-consultation-document | AGS | 04-May-22 | 2021 | UK |
| 217 | Proposal to add folic acid to flour: consultation response > https://www.gov.uk/government/consultations/adding-folic-acid-to-flour/outcome/proposal-to-add-folic-acid-to-flour-consultation-response | AGS | 04-May-22 | 2021 | UK |
| 218 | Providing quality care for women > https://www.rcog.org.uk/media/xt2fqcw0/maternitystandards.pdf | Additional searches | 12-May-22 | 2016 | UK |
| 219 | Psoriasis: assessment and management > https://www.nice.org.uk/guidance/cg153/resources/psoriasis-assessment-and-management-pdf-35109629621701 | NICE | 30-Apr-22 | 2017 | UK |
| 220 | Public Health England – Supplementary written evidence > https://committees.parliament.uk/writtenevidence/656/html | Additional searches | 06-May-22 | 2020 | England |
| 221 | Ready Steady Baby! Alcohol and pregnancy > https://www.nhsinform.scot/ready-steady-baby/pregnancy/looking-after-yourself-and-your-baby/alcohol-and-pregnancy/ | Additional searches | 12-May-22 | 2021 | Scotland |
| 222 | Ready Steady Baby! Health conditions before pregnancy > https://www.nhsinform.scot/ready-steady-baby/pregnancy/health-problems-in-pregnancy/health-conditions-before-pregnancy/ | Additional Searches | 04-May-22 | 2020 | Scotland |
| 223 | Ready Steady Baby! Vitamins and minerals in pregnancy > https://www.nhsinform.scot/ready-steady-baby/pregnancy/looking-after-yourself-and-your-baby/vitamins-and-minerals-in-pregnancy/ | Additional searches | 13-May-22 | 2022 | Scotland |
| 224 | Review of A Strategy for Maternity Care in Northern Ireland (2012-18) > https://www.rqia.org.uk/RQIA/files/3d/3d4d9d13-8079-403f-80d6-ee79008718da.pdf | AGS | 23-Apr-22 | 2017 | NI |
| 225 | Review of Perinatal Mental Health Services in Northern Ireland > https://www.rqia.org.uk/RQIA/files/28/28f4ee85-a5e9-4004-b922-525bc41ae56d.pdf | AGS | 23-Apr-22 | 2017 | NI |
| 226 | Review of Specialist Sexual Health Services in Northern Ireland October 2013 > https://www.rqia.org.uk/RQIA/files/11/1114fc0c-1244-46ba-b32a-6b85d72e7b9e.pdf | AGS | 27-Apr-22 | 2013 | NI |
| 227 | Rheumatoid arthritis & pregnancy > file:///C:/Users/40345946/Downloads/Rheumatoid%20arthritis%20&amp%3B%20pregnancy.pdf | AGS | 11-May-22 | 2019 | UK |
| 228 | Risks and complications of fertility treatment > https://www2.hse.ie/conditions/fertility-problems-treatments/risks-complications/ | AGS | 06-May-22 | 2020 | Ireland |
| 229 | Rubella and pregnancy> https://www.hse.ie/eng/health/immunisation/pubinfo/adult/rubella/engleafrubella.pdf | Additional searches | 13-May-22 | 2020 | Ireland |
| 230 | Safe midwifery staffing for maternity settings > https://www.nice.org.uk/guidance/ng4 | NICE | 02-May-22 | 2015 | UK |
| 231 | Safe Use of Medicines During Pregnancy & Lactation > https://eurohealth.ie/wp-content/uploads/2016/10/Safe-Use-of-Medicines-During-Pregnancy.pdf | AGS | 06-May-22 | 2017 | Ireland |
| 232 | Sapropterin for treating hyperphenylalaninaemia in phenylketonuria > https://www.nice.org.uk/guidance/ta729 | NICE | 02-May-22 | 2021 | UK |
| 233 | Saving Babies’ Lives Version Two > https://www.england.nhs.uk/wp-content/uploads/2019/03/Saving-Babies-Lives-Care-Bundle-Version-Two-Updated-Final-Version.pdf | Additional Searches | 09-May-22 | 2019 | England |
| 234 | Saving Lives, Improving Mothers’ Care > https://www.npeu.ox.ac.uk/assets/downloads/mbrrace-uk/reports/maternal-report-2021/MBRRACE-UK_Maternal_CORE_Report_2021_-_FINAL.pdf | AGS | 23-Apr-22 | 2021 | UK |
| 235 | Scottish Perinatal Mental Health Care Pathways NHS > https://www.pmhn.scot.nhs.uk/wp-content/uploads/2021/06/Care-Pathways-full.pdf | Additional searches | 04-May-22 | 2021 | Scotland |
| 236 | Sexual and reproductive health and HIV: applying All Our Health > https://www.gov.uk/government/publications/sexual-and-reproductive-health-and-hiv-applying-all-our-health/sexual-and-reproductive-health-and-hiv-applying-all-our-health | AGS | 12-May-22 | 2022 | England |
| 237 | Sexual and Reproductive Healthcare programme e-SRH > https://www.e-lfh.org.uk/programmes/sexual-and-reproductive-healthcare// | AGS | 12-May-22 | nd | UK |
| 238 | Shine Thinking about having a baby > https://www.shinecharity.org.uk/folic-for-life/thinking-about-having-a-baby | AGS | 13-May-22 | nd | UK |
| 239 | Sickle cell disease and pregnancy patient information leaflet > https://www.rcog.org.uk/for-the-public/browse-all-patient-information-leaflets/sickle-cell-disease-and-pregnancy-patient-information-leaflet/ | Additional searches | 11-May-22 | 2023 | UK |
| 240 | Sickle Cell Disease in Pregnancy, Management of (Green-top Guideline No. 61) > https://www.rcog.org.uk/media/nyinaztx/gtg_61.pdf | Additional searches | 11-May-22 | 2011 | UK |
| 241 | Smoking and pregnancy patient information leaflet > https://www.rcog.org.uk/for-the-public/browse-all-patient-information-leaflets/smoking-and-pregnancy-patient-information-leaflet/ | Additional searches | 11-May-22 | 2015 | UK |
| 242 | Sodium valproate > https://www.medicinesinpregnancy.org/Medicine--pregnancy/Sodium-valproate/ | Additional searches | 06-May-22 | 2020 | UK |
| 243 | Specialist perinatal mental health services > https://www.hse.ie/eng/services/list/4/mental-health-services/specialist-perinatal-mental-health/specialist-perinatal-mental-health-services-model-of-care-2017.pdf | Additional searches | 12-May-22 | 2017 | Ireland |
| 244 | Spina bifida > https://www.nidirect.gov.uk/conditions/spina-bifida#toc-5 | Additional searches | 11-May-22 | nd | NI |
| 245 | State of Child Health 2020 (Wales)> https://stateofchildhealth.rcpch.ac.uk/wp-content/uploads/sites/2/2020/03/SOCH-WALES-02.03.20.pdf | AGS | 04-May-22 | 2020 | Wales |
| 246 | State of Child Health Report 2017 > https://www.rcpch.ac.uk/sites/default/files/2018-05/state_of_child_health_2017report_updated_29.05.18.pdf | AGS | 23-Apr-22 | 2017 | UK |
| 247 | Statement from the Clinical Effectiveness Unit - Pre-conception Care FSRH > file:///C:/Users/40345946/Downloads/pre-conception-care-edit-15-9-16.pdf | AGS | 13-Jun-22 | 2016 | UK |
| 248 | The benefits of exercising/being active when trying to conceive > https://www.tommys.org/pregnancy-information/planning-a-pregnancy/are-you-ready-to-conceive/being-active-when-trying-conceive | Additional Searches | 09-May-22 | 2021 | UK |
| 249 | The folate status of pregnant women in the Republic of Ireland; the current position > https://www.hse.ie/eng/about/who/acute-hospitals-division/woman-infants/national-reports-on-womens-health/folate-status-in-pregnant-women-in-republic-of-ireland.pdf | Additional searches | 12-May-22 | 2017 | Ireland |
| 250 | The Food Foundation’s Early Years Nutrition Study > https://foodfoundation.org.uk/sites/default/files/2021-12/EARLY%20YEARS%20CONCEPT%20NOTE.pdf | AGS | 23-Apr-22 | 2021 | UK |
| 251 | The health and wellbeing of children in the early years >https://childrensalliance.org.uk/wp-content/uploads/2021/10/WG1-EarlyYears-Oct2021.pdf | Additional searches | 11-May-22 | 2021 | UK |
| 252 | The NHS Long Term Plan > https://www.longtermplan.nhs.uk/wp-content/uploads/2019/08/nhs-long-term-plan-version-1.2.pdf | AGS | 10-May-22 | 2019 | England |
| 253 | The Perinatal Mental Health Care Pathways > https://www.england.nhs.uk/wp-content/uploads/2018/05/perinatal-mental-health-care-pathway.pdf | Additional searches | 12-May-22 | 2016 | England |
| 254 | The UK Strategy for Rare Diseases > https://assets.publishing.service.gov.uk/government/uploads/system/uploads/attachment_data/file/260562/UK_Strategy_for_Rare_Diseases.pdf | Additional searches | 04-May-22 | 2013 | UK |
| 255 | Thinking of having a baby to help you & your baby – now or in the near future? > https://www.contraceptionchoices.org/sites/default/files/media/WomensHealthA54pp%20Bro%20AW.pdf | AGS | 10-May-22 | nd | England |
| 256 | Treatment -Behçet's disease > https://www.nhs.uk/conditions/behcets-disease/treatment/ | Additional searches | 13-May-22 | 2019 | UK |
| 257 | Treatment -Polycystic ovary syndrome > https://www.nhs.uk/conditions/polycystic-ovary-syndrome-pcos/treatment/ | Additional searches | 13-May-22 | 2022 | UK |
| 258 | Trying to get pregnant > https://www.nhs.uk/pregnancy/trying-for-a-baby/trying-to-get-pregnant/ | Additional searches | 12-May-22 | 2020 | UK |
| 259 | Types of fertility problems > https://www2.hse.ie/conditions/fertility-problems-treatments/ | AGS | 06-May-22 | 2019 | Ireland |
| 260 | Update Report on Folic Acid and the Prevention of Birth Defects in Ireland > https://www.fsai.ie/publications_folic_acid_update/ | AGS | 06-May-22 | 2016 | Ireland |
| 261 | Vaccines you need before you get pregnant> https://www2.hse.ie/wellbeing/pregnancy-and-birth/trying-for-a-baby/vaccines-before-pregnancy/ | Additional searches | 13-Jun-22 | 2022 | Ireland |
| 262 | Valproate in women and girls who could get pregnant > https://www.hse.ie/eng/services/publications/mentalhealth/valporate-in-women-and-girls-who-could-get-pregnant.pdf | Additional searches | 12-May-22 | 2019 | Ireland |
| 263 | Valproate in women and girls who could get pregnant > https://www.rcpsych.ac.uk/mental-health/treatments-and-wellbeing/valproate-in-women-and-girls-who-could-get-pregnant | Additional searches | 30-Apr-22 | 2018 | UK |
| 264 | Vitamins, supplements and nutrition in pregnancy > https://www.nhs.uk/pregnancy/keeping-well/vitamins-supplements-and-nutrition/ | Additional searches | 13-May-22 | 2020 | UK |
| 265 | We are Croydon - Early Experiences Last a Life time - The first 1000 days from conception to the age of 2 > https://www.croydon.gov.uk/sites/default/files/articles/downloads/Director%20of%20Public%20Health%20report%202018.pdf | Additional searches | 12-May-22 | 2018 | England |
| 266 | When to see your GP about fertility problems > https://www2.hse.ie/conditions/fertility-problems-treatments/when-see-gp/ | AGS | 06-May-22 | 2019 | Ireland |
| 267 | Withdrawal of, and alternatives to, valproate-containing medicines in girls and women of childbearing potential who have a psychiatric illness > https://www.rcpsych.ac.uk/docs/default-source/improving-care/better-mh-policy/position-statements/ps04_18.pdf?sfvrsn=799e58b4_2 | Additional Searches | 13-May-22 | 2018 | UK |
| 268 | Women and Alcohol - What every woman needs to know. > https://www.alcohol-focus-scotland.org.uk/media/60091/Women-and-alcohol-leaflet.pdf | AGS | 04-May-22 | nd | Scotland |
| 269 | Women and Families Maternal Mental Health Pledge > https://www.nhsinform.scot/care-support-and-rights/health-rights/mental-health/women-and-families-maternal-mental-health-pledge/ | Additional Searches | 04-May-22 | 2020 | Scotland |
| 270 | Women With Diabetes > https://www.qub.ac.uk/elearning/public/WomenWithDiabetes/WhyPlan/ | AGS | 12-May-22 | nd | NI |
| 271 | Women’s Health Plan A plan for 2021-2024 > https://www.gov.scot/binaries/content/documents/govscot/publications/strategy-plan/2021/08/womens-health-plan/documents/womens-health-plan-plan-2021-2024/womens-health-plan-plan-2021-2024/govscot%3Adocument/womens-health-plan-plan-2021-2024.pdf | AGS | 04-May-22 | 2021 | Scotland |
| 272 | Women's health: migrant health guide > https://www.gov.uk/guidance/womens-health-migrant-health-guide | Additional searches | 12-May-22 | 2021 | England |
| 273 | Written Statement: Government’s decision to introduce mandatory fortification of flour with folic acid to help prevent foetal neural tube defects > https://gov.wales/written-statement-governments-decision-introduce-mandatory-fortification-flour-folic-acid-help | AGS | 04-May-22 | 2021 | Wales |
| 274 | Your guide to pregnancy and fertility in thyroid disorders > https://www.btf-thyroid.org/Handlers/Download.ashx?IDMF=941f817e-a8da-4ea9-8ee9-83772477c280 | Additional searches | 12-May-22 | 2018 | UK |
| 275 | Your sexual health Where to go for help and advice > https://www.unidocs.co.uk/docs/fpa/your-sexual-health-where-to-get-help-and-advice.pdf | AGS | 25/04/2022 | 2012 | UK |
| 276 | Zika virus > https://111.wales.nhs.uk/encyclopaedia/z/article/zikavirus/ | Additional searches | 12-May-22 | 2021 | Wales |
| 277 | Zika virus and pregnancy > https://www2.hse.ie/conditions/zika-virus-pregnancy/ | Additional searches | 13-May-22 | 2021 | Ireland |
| 278 | Zika virus in pregnancy > https://www.medicinesinpregnancy.org/Medicine--pregnancy/Zika-virus/ | Additional searches | 06-May-22 | 2017 | UK |

Abbreviations:

AGS: Advanced Google Search; NI: Northern Ireland; UK: United Kingdom

# Additional file 7

Table 1. List of healthcare professionals and services mentioned as those who could optimise preconception care.

| ***Healthcare professionals mentioned*** |
| --- |
| General practitioners, nurses, pharmacists, midwives, health visitors, obstetrics, gynaecologists, social workers, paediatricians, psychologists, psychiatrists including addiction psychiatrists, laboratory staff, biochemists, neurologists, nephrologists, diabetes, epilepsy and hypertensive disorders specialists, cardiologists, geneticists and genetic counsellors, chest physicians, rheumatologists, gastroenterologists, haematologists, oncologists, radiographers, specialists in sexual and reproductive health and sexually transmitted infections, fertility specialists, pelvic floor clinicians, dentists, anaesthetists, optometrists, physiotherapists, dietitians, occupational therapists and other Allied Health Professionals. |
| ***Other services and settings mentioned*** |
| Maternity services, weight management services, migrant health-related services, specialist drugs and alcohol services, smoking cessation services, clinical Pilates providers, education services and higher education establishments, teaching hospitals, food industries, the employment sector and workplaces, voluntary organisations and non-clinical community-led services (e.g., debt management, legal advice, arts activities). |
